# Supplementary material for: Transcriptomes reveal microRNAs and mRNAs in different photoperiods influencing cashmere growth in goat
Source: PLoS One. 2023 Mar 17;18(3):e0282772. doi: 10.1371/journal.pone.0282772 (PMC10022811; doi:10.1371/journal.pone.0282772)

**Construction of Dual Luciferase Reporter Vector**

**1 Instruments and reagents**

**1.1 Instruments**

| Instrument Name | Instrument model | company |
| --- | --- | --- |
| Gel imaging system | ChemiDoc XRS^+^ | Bio-RAD |
| Electrophoresis system | POWER/PAC300 | Bio-RAD |
| Electrophoresis tank | JY-SPCT | Junyidongfang,Beijing |
| Low temperature centrifuge | 5417R | Eppendorf |
| High-Pressure Steam Sterilization Pot | YM75 | Sanshen,Shanghai |
| Ultramicro Spectrophotometer | Nanodrop 1000 | Thermo |
| Pipettor | 2.5µL、10µL、200µL、1000µL | Eppendorf |
| PCR amplifier | Veriti | ABI |
| Temperature Incubator | 303-00AB | Kuntian,Shanghai |
| Thermostatic oscillator | THZ-B2A | Saidelisi,Tianji |
| Electronic scales | BS-224S | Sartorius |
| Microwave oven | P70D20TL-D4 | Galanz |

**1.2** **Experimental reagent**

| **Reagents name** | Article Number | manufacturer |
| --- | --- | --- |
| Agarose | 111935 | BioWest |
| PrimeSTAR® HS DNA Polymerase | R010A | TAKARA |
| TaKaRa Ex Taq® | RR001A | TAKARA |
| dATP | 4026Q | TAKARA |
| 6×Loading Buffer | D1010 | Sigma-Aldrich |
| Trans2K^®^ Plus DNA Marker | BM111-01 | TransGen Biotech |
| Trans DNA Marker Ⅱ | BM411 | TransGen Biotech |
| NheI-HF^®^ | R3131S | NEB |
| XhoⅠ | R0146S | NEB |
| GelStain | GS101-02 | TransGen Biotech |
| Ampicillin | ST007 | Beyotime Biotechnology |
| Kanamycin | ST101 | Beyotime Biotechnology |
| T4 DNA ligase | M0202V | NEB |
| Trans5α Chemically Competent Cell | CD201-01 | TransGen Biotech |
| Yeast extract | 01-012 | AOBOX |
| NaCl | GB/T1266-2006 | GUANGFUBIAOWO |
| Peptone | 01-002 | AOBOX |
| agar | Q/HG 3554-99 | Zhiyuan,Tianjin |
| Agarose Gel Recovery Kit | 9762 | TAKARA |
| Blood/Cell/Tissue Genomic DNA Extraction Kit | DP304 | TIANGEN BIOTECH |
| Plasmid microextraction kit | DP103-03 | TIANGEN BIOTECH |
| Absolute ethyl alcohol | 10009218 | Sinopharm |
| Primer |  | GENEWIZ |
| RNase/DNase-free ddH_2_O | RT121-02 | TIANGEN BIOTECH |

**1.3 Genome extraction**

Genome was extracted from the samples stored in the -80℃ freezer following the kit procedure (TIANGEN BIOTECH, China).

**2 DNA testify**

**2.1. DNA purity and concentration**

DNA purity and concentration were detected using 2μL extract, using NanoDrop2000 to measure the purity and concentration of DNA, the results are shown in the table below:

**2.2 Agarose gel electrophoresis**

| **NO** | **Origin** | **Sample type** | **concentration（ng/ul）** | **OD260/280** |
| --- | --- | --- | --- | --- |
| 1 | Cashmere goat | blood | 47.2 | 1.98 |


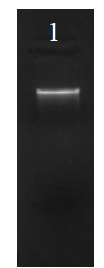
DNA integrity was determined by 1.5% agarose gel electrophoresis and 2μL was taken. The results are shown in the figure below:

**3 Sequence information**

**3.1 Target sequence**

3.1.1 XM_005676701.3

The gray marker is the CDs，the unmarked area is UTR，the red marker is the site of action，the yellow marker is the protective base，and the blue marker is the restriction site.

GTCGGAAGTCAAAGGTCAGTAAATAGTGGTGATGTCATGCGGGCAAGATGGCGGAAGGGGAGGACGTGGGATGGTGGCGGAGCTGGCTGCAGCAGAGCTACCAGGCAGTCAAAGAGAAGTCCTCCGAAGCCCTGGAGTTCATGAAGCGGGACCTGACGGAGTTCACCCAGGTGGTGCAGCGCGACACGGCCTGCACCATTGCGGCCACCGCCAGCGTGGTCAAGGAGAAGCTGACTACTGAAGGCTCCTCTGGAGCGACAGAGAAAATGAAGAAAGGGCTGTCTGACTTCTTGGGGGTGATCTCCGATACCTTTGCTCCCTCACCAGACAAAACCATCGACTGTGACGTCATCACCCTGATGGGCACACCTTCTGGCACGGCTGAGCCCTATGATGGCACCAAGGCTCGCCTCTATAGCCTGCAGTCAGACCCAGCAACCTACTGCAATGAACCAGATGGGCCTCCAGAGTTGTTTGATGCCTGGCTTTCCGAGTTCTGCTTGGAGGAGAAGAAGGGGGAGATCTCAGAGCTCCTTGTAGGCAGCCCCTCCATCCGGGCCCTCTACACCAAGATGGTGCCTGCAGCTGTTTCCCATTCAGAATTCTGGCATCGGTATTTCTATAAAGTCCATCAACTAGAGCAGGAGCAGGCCCGGAGGGATGCCCTGAAGCAGCGGGCAGAACAGAGCATCTCTGAAGAGCCTGGCTGGGAGGAGGAAGAAGAGGAGCTTGCGGGCGTTTCACCCACATCCCTGAAAGAGGCAAAGATTCCTGTGGCCAGACCTTCCACATCGCCTGAAGGAGGACCTGGTCCCCAGAGCCCCTGCGAAGAGAATCTGGTGACCCCCGTTGAACCTCCAACAGAGGTGACCCCCTCGGAGAGCAGTGAGAGCATCTCCCTGGTGACACAGATTGCCAAACCGCCCCCTGCCTCTGAGGCCCCAGCACTGCCCAAGGACCTGTCCCAGAAGCTTCTAGAGGCATCTTTGGAGGAACAGGACCTGGCTGTGGATACGGGCGAGACTGGACCCCCACCCCGAGCTCAGTCTAAGCCCCACACCCCTGCTTGCCGCCCCAGCGGTCCAGAGCCCCGGCCTCCAGCCAGAGTAGAGACTCTCAGGGAAGAGGTCCTCACAGACTTACGGGTGTTTGAGCTGAACTCGGACAGTGGGAAGTCTACACCCTCCAACAATGGGAAAAAAGGCTCGAGTACAGACATCAGTGAGGACTGGGAGAAGGACTTTGACTTGGACATGACTGAAGAAGAAGTGCAGATGGCGCTTTCCAAAGCGGACGCCTCCGGTGAGCTGGAAGACGTAGAGTGGGAGGACTGGGACTGAGGGGAGCCAGAGCAAGCAGCTCCCCCACCCACAGCACTTCCCACTTCCCTCGCCCGTCTCAGCCCGGCCCCGGAAGACACTGACTAAGAATGTCCCCCAAATGGCCTCTGTCAACCAGAGCTCTTGGCAGATTCTGGGTTGTTCCTTGTTGGCCCTTCGGGCCTCTGCTCACGTCTGGGAAGGGGCTCGCTTAATCCAAACCAGGAACTCTGACTTGTGCCAACCATAGGATGACCTGAGGGGGAGGAAACTTCCCCCACCCACCCCACCCCCCAAAGAAGAGCCTACATTTCTCTGCTGAATACCCACTGTTCCTGGGGACTCCTGCTGAGGTGTCCCAAGGGATAGCCCTCGCCCATGTGCCTGTGTAGACAGAGGCTAAACCACCGGTCTCATGGAGGATGCCGAGACAACACTGTGTCACCCATGAGCCGGCAAGGAAGGCCGTGCCACCTGCCCTTGGCTTGACAGAGATGGCAGACACACTTTGGTTCCTATCCCAGCGGGTAAGAGGCATTCATTTTTGGGAAAGTTGCCTCCCTTAGGAATCTGTCCCTCCCAGGCATTTTCCATTCCAGGAAGGGCTCCTTGTGGTTCAGAATCTAGAGACCAAACTCACTCCTTTCCCCCAGTCCAGGCTGGTATGTCCCCAGCACCTTTCCCAAGCCGTCTTCATGTCAGATGCACCCGAGTCCTTAGCCCAGCTGTGCCACCTGCAAGAGTCTGCTCTTGCCCTTCTTCCCCTCCCCAAGAAGGGAGGGGGCCACTTCAGGCCCTTCCGTGTGTTGTCTGGCGGGGTACCTTGTCCAGCCAGCCACCCACTTTGACTCCCCCGTAGCTTAGGACACAAGCCAGCTACCAGCGGTACAGAGCAGTGATCAAAGCCGAGTACTTACAACTCTGGTAAGCCCAACTTCTCCGCCTCAACCCTTCTGCCTCTTGGAGGGATACGCTGGGGGTGAGCTGCTTGAGATTCTCGACAGGCTTCTGCAAAAGCTCTTCCCTCCTGAAGGCAGATCCAGTCTTGGTGGCTCTCACCCTCCACGCTGGTAAAGCTGCACCTCTCTTGGGGGGACGAGGGGCTGCAGGAATCCCTGGAGACCCTGGTGCTTCACGATGCTGCTCCGGTGATTCTTGTACATAATCTGGTGTGTTCACCAATGATTTAAAGGGATCGTGGTCAGGGACGCCAAGAGAGTGGTGATCACTTCCACTTCAAACCTTCAATGAGGGGGTGGGATGGAGAGAATGCTGAATCTTTTTTTTTTCTTTTCTTTTTCATGGGATAGGATTTTTCTCTTTGTAATTATTTCTTTAGTTTAATTAACCTTTTGGTTGTTTGTGCAATATTATATATTTTAAATTATAATGTATCTCCCCAGAGTATTTTGTAGCTGGGATAAGAAAAAAGGAAAAAAAAAAAAAAAAAGACTCTAACAGCTGTTAGTTTTGTAATTAAAAAAGAAAGAAAAAAGAACTTTGTCCTGAACCTTTTCCAGACTTGCCGTTAACAGCTATTAAAGAGATTCAACAGAAGCTGGAAGGTGTCTGGGGCTCTGTTCTTTTCCCTTACAGCAGCTGCCCCAGAGCCAGGGCAGGACAGGGTGTGCAGGGTGCCCTCCTGCTTGGAGCCTGGGGGAGGGGTCAGGATGGGAGGTAGTAGCCTGCATTCCAAGGAGGTCCCTTGTCTTCATCCCAGACTCACCTTGGCCTTTGGGGTGAGGAAGGGACAGGGAGGGCCCCCAGAAGGCCTCTCACCAAGACAAGGAGTCAAGAGGCCGTTTCTGCATTCCCAACGCTTCTGCTTTTATTTGGAAAGGAAGGTGCAACAACAGTGGTTCCAACTGTTCTTGGTTATTTTCCTCTCCTCCCCCTGTATGTGTATGATTCTGGGGTCTATTTTTAAGTAACTTTAGTTAAACACCTGTATGAAAATCACTTGTCAGCCATAATAAAAGGGAAGTCTGAATGAG

WT: ACGATGCTGCT

MU: CATCGTAGTAG

F: CTAGCTAGCTACCTTGTCCAGCCAGCCACCC

WT-R: CCGCTCGAGAGCAGCATCGTGAAGCACCAGG

MU-R: CCGCTCGAGCTACTACGATGGAAGCACCAGG

Amplification product size:333bp.

3.1.2 XM_018064739.1

GACTCGGGGCTGAGCACAGCTGGCTGGGCGTCGGCCGGGCAGTTGGCGTCGGCTGAGTCGGCGCGTCGGGCACTGAGGACCGGCCATGGAGCGCGAGGTCCAGCGGGTTCGCGCGGCTTTCGGCTCCGGCCGCTCTCGCCCCTTGACGTTCCGGCGGCGGCAGCTCGAGGCCCTGCGCGCGATGGTGCAGGAGCGCGAAAAAGACATCCTGGCGGCCATCGGTGCGGACCTGAGCAAGAGCGAATTCAACGCATACAGTCAAGAAGTCATTTCTGTGCTTGGAGAAATTGATCTCGTGCTGGAGAAGCTTCCGGAATGGGCTGCTGCTAAACCAGCTCAGAGGAACCTGCTCACCATGCTGGACGAGGCCTACATCCAGCCAGAGCCCCTGGGGGTCGTCCTGATTATTGGAGCTTGGAACTACCCCTTTGTCCTCACCATCCAGCCCCTGATAGGAGCCATCGCTGCAGGAAATGCTGTGATTATCAAGCCTTCCGAAGTAAGTGAGAATACAGCCAAGCTCTTGGCTAAGCTCCTCCCTCAGTACTTGGACCAGGATCTGTACGCCGTCGTCAATGGTGGTGTTGAGGAGACCACGGAGCTTCTGAAGCAGCGATTTGACCACATTCTCTACACGGGAAACACCACTGTTGGAAAAATCGTCATGCAGGCTGCTGCCAAGCATCTGACCCCTGTGACCCTTGAACTGGGAGGAAAGAGCCCATGCTTCGTGGACCGAGACTGTGACTTGGATGTCGCCTGCCGACGCATCGCCTGGGGCAAGTTCATGAACTGCGGTCAGACCTGCATTGCCCCCGACTACGTGCTCTGTGAGCCGTCCCTCCAGGACCAGATCGTGCAGAAGGTGCAGGAGGCCGTGAAGGAATTTTATGGAGAAAATGTAAAAGAATCTCCCGACTACGAGAGGATCGTCAATCTTCGTCATTTTAAGAGGATACAGAGTCTGCTGGAAGGACAGAAGATAGCTTTTGGTGGGGAGATGGATGAGGCCACACGCTACATAGCCCCAACAGTACTCACTGATGTGGATCCTGACACCAAGGTGATGCAGGAAGAAATTTTTGGACCAATTCTTCCAATAGTGCCTGTGAAGAATGCAGATGAAGCCATACAGTTCATAAATGAACGCGAAAAGCCCCTAGCTTTCTATGTGTTTTCTCACAACAGTAAGCTCATCAAGCGGATGATCGACGGGACGTCCAGCGGCGGTGTCACAGGCAATGACGTCATCATGCACTTCATGCTCAGCTCTCTGCCCTTCGGAGGCGTGGGGTCCAGCGGGATGGGAGCGTATCACGGAAAACACAGTTTCGATACTTTTTCCCATCAACGTCCCTGTTTATTAAAAACTCTCAAGCGGGAAGGCGCGAACCAACTCCGGTACCCTCCCAACAGCCAGTCGAAGGTGAACTGGGCGAAATTTTTCTTGTTGAAGCGGTTCAACAAAGGGAAACTCGGCCTCCTGCTGCTCACTCTCCTGGGCATCCTGGCCGCCGTGCTTATCAAGGGCGGATACTACTGATGGACCCTAGCCACCTCCTCATCGTCTCTGCTGAATTATTATTCTGCTCAGTGGCTAACCAACCAATCATTTTTAAATTGTACCAGAACTTTTTAAGAAGATATGCAAACGCACCATCCTCGCCATTCACCATTAATAAAACTCACTGCTTCAGCCAAAGTCCCCCACTTACCTCCCCACCCCGGCCCCCTAGGACTGAGCACGCTCACTTGGAGAACTTGCATCCCTAGAGCTCTGGGCAGGGACAGGGAGCGCACTGGGTGAGCCCACATTCCCAGACCTGCTCAGTGACTCTTTACCAGGGACATGGGGCTTGCCACCCCCACCCCCACCTTCAGGCAGCACTCCCTGAAGCCTTCCAGAGACTGCCCTGCACATGTTTCCAGGTCTCAGTCTAGAGAGGTGAGGCGAGATCTCCCAGAGTCCTCAGGGTTCAGGGCACTAGAACTGGGTTGGGGGGGGGCCCTGTCCCTGATCCTGGGACCAGAAGACAGCTCCAGGAGGCAGCCCTCCTTTCTCACCACTCTCTGTCCTGTCCTGAGCTCGGCTTGTGACGGTCCGTTGTGATGTCCACACATGGTAAATCCTCGGGCTCCTGGAAGGCAACTGCCAGTTTGGTGCAAGAATCATCTGTCATTCGTAGACTTTGTGCGTCTTACAAAGTCACTGGCCTGTTTCACTGCTATCGTCATGGTACTAAGACGATACCCACCCTTGATCAGAGGCTGCACATGGCAAGTCAGTGGCTCTTGAGGGACAAATCTTCGTTTCCTGCTTCTGTACCACAAGGCGAGTAAGCATCCCCTTGGCCTCCCTTGTGGCTCAGCTGGTAAAGAATCCGCCTGCAATGTGGGAGACCTGGGTTGGGAAGATCCCCTGGAGAAGGGAATGGCTACCCACTCCAGTATTCTGGCCTGGAGAATTCCATGGTCTCTGTAGTCCATGGGGTCACAAAGAGTCGGACACAACTGATCGACTTTCACTTTTGGAAACCTGAGAGGGGCATTGCTGAACGGATTCTGTCCAGAACGTAACGTGGGGAGCAGTTTCTGTCCTGGACAAGAGGTCTTGGGCGTTCACAACTTACAGCCTGAGCCCCAGGGGCTGGTGAGGGGCATCTGGAAGAGGGCTCTCCATGACCAGGACAGCAGCCAGCCCACTGAAGGGGACCTCCCAAGCACAGTTGGGACCAGACCCAGCACTTCAGCCAAACAGTCTCAAGAAGAGCCCTGGGGGTCCTCCCTGCCCCTACACTCAATGAGAGAATGTACCTCACCTCACAGCACGTGATCCACCATTGAATGAACCTGCTTTGGCTGACTGCAGAGCGTTACGCCTTCATTACAGAACACAGGGATCAACAACCAATCACTAGGCTGGCTTAGGACATACCTGCTTACACTGGGGCTTTATCAGAATAATCAGCCATAAAATATCTCAGACAATGGAGTTTTCATTTGGTATATTGTGCATTTTACTTTCTAATTAAAATTATTTGCTTGTCTACTGGCA

WT:GCCTCCC

MU:TAAGAAA

F:CTAGCTAGCAGCCCTCCTTTCTCACCACTCTCT

WT-R:CCGCTCGAGACAAGGGAGGCCAAGGGGAT

MU-R:CCGCTCGAG TTTCTTACAAGGGGATGCTTA

Amplification product size:332bp.

3.1.3 XM_018063741.1

GCCACTACCCAGCGGCCGGCCGGCGCAGCTAGGAGCTGACTCCCGGGGACTTGGGGCCGCAGGGAGCCACCTCTCCACAGAGCTGAAACTTGACCCCAGAGCATCTCTGCAGAGTGAGGAAAGGACTAGAACATGCTCTAAAGTCCTCAGCAACAAGACCCAGGGAACCACAAGCTCAGCCCACAGCCTGAAGCTGCCCTGCCCCCTTGCCCACCATGGCCTCCGATGACAAGAATGGTGAGAGGGTCTCCTCGGTGTCCAGCAGCCGCCTGCAGAGCCGGAAGCCGCCCAACCTGTCCATCACCATCCCTCCGCCTGAGACCTCGGCCCCGAGCGAGCAGGCCAGCATGCTGCCCCAGGAACCCGGAGGGCAGAGGCCCAGGAACCCGGCCCTCCTGAAGAGCGTCAGCCTCCAGGAGCCGAGAGGGCGATGGCAGGAAGGTGGCCCGGAGAAGCGTCCTGGCTTCCGCCGCCAGGCCTCGCTGTCCCAGAGCATCCGCAAGGGCGCGGCCCAGTGGTTCGGGGTCAGCGGAGACTGGGAGCTGAAGCGGCAGCACTGGCAGCGCCGGAGCCTTCACCACTGCAGCGTGCGCTACGGCCGGCTCAAGGCCTCATGCCAGCGGGACCTGGAGCTCCCCAGCCAAGAGGTGCCCTCCTTCCAGGACACCGAGTCCCCAAAGCCCTGCAAGATGCCCAAGATCGTGGACCCCCTGGCCCGAGGACGGGCATTCCGCCACCCGGAAGAGGTGGAACGGCCCCATGCCCCACACCCACCGCTGACGCCCGGGGTCCTTTCCCTCACGTCCTTCACCAGCGTCCGCTCCGGCTATTCCCACCTGCCCCGCCGCAAGAGGATCTCTGTGGCCCACATGAGCTTTCAAGCTGCTGCAGCCTTGCTTAAGGGGCGCTCGGTGCTGGAGGCCACGGGACAGCGGAGCCAGGTGGTCAAGTGCAGCTTTGCTTACCCCAGCTCCCTGGAGGAGGATGCGGTCGACGGGGCAGAGACATTCGACTCCTCGTTTTTTAGTAAGGAAGAAATGAGCTCCATGCCCGATGACGTGTTTGAGTCGCCCCCACTCTCGGCCAGCTACTTCCGGGGGATCCCACACTCAGCCTCCCCAGTCTCCCCTGATCAGATCCCCCTGAAGGAGTCCAGCCGAGCCCCAGTGCCCCCCACCAAACGCGGCAAGCGCATCGCCTCCAAGGTGAAGCACTTTGCCTTCGACCGGAAGAAGCGGCACTACGGCCTGGGCGTAGTGGGCAACTGGCTGAACCGCACTTACCGCCGCAGCATCAGCAGCACAGTGCAGCGGCAGCTGGAGAGCTTCGACAGCCACCGGCCCTACTTCACCTACTGGCTGACCTTTGTCCACATCATCATCACGTTGCTGGTGATCGGCACGTATGGCATCGCTCCCGTGGGCTTCGCCCAGCACATCACCACCCAGCTGGTGCTCAGGAACAAAGGTGTGTATGAGAGCGTGAAGTACATCCAGCAGGAGAACTTCTGGATCGGCCCCAGCTCGATCGACTTGATCCACCTGGGAGCCAAGTTCTCACCTTGCATCCGGAAGGACCAACAGATCGAGCAGCTCGTGCTGCGGGAGCGAGACCTGGAGCGGGACTCGGGCTGCTGTGTCCAAAACGACCGTTCGGGCTGCATCCAGACCCAAAGGAAGGACTGCTCGGAGACTCTGGCCACTTTTGTCAAGTGGCAGGATGATACGGGGCCCCCCATGGACAAGTCTGATCTGGGCCAGAAGCGGACTTCTGGGGCCGTGTGCAACCAGGACCCCAGAACCTGCGAGGAGCCGGCCTCTAGTGGTGCCCACATCTGGCCCGACGACATCACCAAGTGGCCGATCTGCACAGAGCAGGCCAAGAGTAACCTCTCGGGCTTCCCGCACATGGACTGCCAGATCCGAGGCCGCCCCTGCTGCATCGGCACCAAGGGCAGCTGTGAGATCACCACGCGCGAATACTGTGAGTTCATGCACGGCTATTTCCACGAGAAGGCCACGCTCTGCTCCCAGGTGCACTGCTTGGACAAGGTGTGTGGACTGCTGCCCTTCCTCAACCCTGAGGTCCCCGATCAGTTCTACAGGCTCTGGCTGTCTCTGTTCCTCCACGCTGGCGTGGTCCACTGCCTTGTGTCCGTGATCTTCCAAATGACCATCCTGCGTGACCTGGAGAAGCTGGCCGGCTGGCACCGCATCTCCATAATCTTCATCCTCAGCGGCATCACCGGCAACCTCGCCAGCGCCCTCTTCCTCCCGTACCGGGCAGAGGTGGGCCCTGCAGGGTCGCAGTTCGGCCTCCTGGCCTGCCTCTTCGTGGAGCTCTTCCAGAGCTGGCAGCTACTGGAGCGGCCCTGGAAGGCCTTCCTGAACCTGTCGGCCATCGTGCTGTTCCTCTTCATCTGCGGCCTCCTGCCCTGGATCGACAACATCGCCCACATCTTCGGCTTCCTCAGTGGGCTGCTGCTGGCCTTCGCCTTCCTGCCCTACATCACCTTCGGCACGAGTGACAAGTACCGCAAGCGCGCCCTCATCCTGGTGTCACTGGTGGTCTTCGCCGGCCTCTTCGCCTCGCTGGTCATCTGGCTGTACGTCTACCCCATCCACTGGCCCTGGGTCGAATACCTCACCTGCTTCCCCTTCACCAGCCGCTTCTGCGAGAAGTACGAGCTGGACCAGGTGCTACACTGACCACCTGCCCGGTGGGGCCGGCGTGGCTGCTCCCACCCAGCTCGGAGGATGCGGGGACTGTGTCTGGACCGAGCTGTGCACTTGCACAGCACCCTGCCCACACCCCAGAGACCCCATGGGGCTGGCCCTGCTCCCAGGAGCTCCCTCCCCCAGGAGAGGCCAAGTCCGAGGGGAGACTCTCCTAAAGGTGGGCTTCTTGTGGGGCCGAGGCCCCCTTCCTCAGGCCTGGGCTTGGGGACACTCGGAGCCTGTACATCTCTGCAGCTCGGGGCCCAGGCCCCGACAGACCTCCACTGCCCGGCCGTCGGGACCCCTCCTGGTCCTGGGATGCTGCTCAGCCCCTACCTCCGGCTCTGCTAGTACGGTCCCCACCCCGCCCCATTACTGTGAGCTAGCTCCTCCTAGGGATGAGGCTGGGGTTCCTGCCAGGTTCCCAGCCCTGCCTGGCCACGGCCCCCCTGCCTGTTGTCTGTCCCCTCTCTGCCCTGTGAGTCCATGCCCAGCTTCCATCAGCGGCTTGGTGAGCCTCCCCATTACGCTCCTGGACCAGACCTGGTGGGAGAGGCTTTGGCCATGGTCTGGGACAGAGAAGGAGCTAAGAGACGCAGCTGGACTGGGAAGCTGAGAGCCTGGGCTGTTACCCAATAGAAGCTAAGCCCACCAGCCCACCCCAGGGCCTCCAGGTCCTCCCAGCCCTCCCAGCCAGCCCTTCCCCTCGAGTTAGTGTTCAGCTTCCTGGGGCTGGACTGATGCCAACCAGCTCACCACCCTCTGCCTCACTTCTGCCAAGAAAGGGGCAGGGCCACCCTGTGGATCTGCGCCCAGGGGTTGCCCAAGGTCAATCAGGACTTTGTTTTCTTAACCAATGTATATCATGGTCGTAATTTTTTAAAGTAACGCTAACTTTGTATGGACAACGTCTGAAATGAATTAAATGATATTCTTTAAGATAGGA

WT:GATGCTGCT

MU:TCGTAGTAG

Primer:

F:CTAGCTAGCCCTGCCCACACCCCAGAGACCC

WT-R:CCGCTCGAGGGCTGAGCAGCATCCCAGGACC

MU-R: CCGCTCGAGCTACTACGACCAGGACCAGGAGG

Amplification product size:261bp

3.1.4 XM_005701596.3

GGCGGGGCCAGGCCCGGCCGAGGCGGAAGCGCCCGAGGAGGGCCCGGCTTCCCGGTGTCCCGCACTTACCCCCTCCTCGGCCCTGAACTGCTTTTGTCCCGCTTCGTGGGGCCCCTCGGGCCTGTTTGTGCGGTGAGCCATGGAGGCTCCGTGGACCCTCACCCTGACCTTGGCCGCAGGCCTGGCTGCTGCCAGCCCACCTAACATCCTGCTGATCTTTGCTGATGACCTGGGCTACGGGGACCTGGGCTCCTATGGGCACCCCAGTTCCACCACCCCCAATCTGGACCAGTTGGCCGCAGGGGGTCTTCGGTTCACAGACTTCTATGTGCCTGTGTCTCTGTGCACACCCTCCCGGGCTGCCCTCCTGACCGGCCGACTCCCAGTTCGGATGGGCTTGTACCCTGGAGTTCTGGAGCCCAGCTCCCGAGGGGGCCTGCCCCTGGAGGAGGTGACCTTGGCTGAGGTCCTGGCTGCCCAAGGCTACCTCACAGGGATAGCTGGCAAGTGGCACCTTGGGGTGGGGCCTGAGGGGGCCTTTCTGCCCCCCCACCATGGCTTCCATCGATTCCTGGGCATCCCGTACTCCCATGACCAGGGCCCTTGCCAGAATCTGACCTGCTTCCCGCCGGCCACCCCCTGCGAAGGCATCTGTGACCAGGGGCTGGTCCCTATCCCCCTGCTGGCCAACCTGTCGGTGGAGGCACAGCCCCCTTGGCTGCCTGGACTCGAGGCCCGCTACGTGGCTTTTGCCCGTGACCTCATGACTGATGCCCAACGCCAAGGCCGCCCGTTCTTCCTGTACTATGCCTCCCACCATACCCACTACCCCCAGTTCAGTGGGCAGAGCTTTCCAGGGCACTCAGGCCGAGGGCCGTTTGGGGACTCCCTGATGGAGCTGGATGCGGCTGTGGGGGCCCTGATGACAGCTGTGGGGGACCTGGGGCTGCTCGGAGAGACACTCGTCTTCTTCACTGCGGACAACGGACCTGAGACGATGCGGATGTCCCACGGTGGCTGCTCTGGCCTCCTGCGATGCGGAAAGGGAACCACTTTCGAAGGGGGCGTCCGAGAGCCCGCCTTGGCCTTCTGGCCTGGCCACATCGCTCCCGGTGTGACCCATGAGCTGGCCAGCTCCCTGGACCTGCTGCCCACCCTGGCAGCCCTGGCGGGGGCCCAGCTGCCCAATATCACCTTGGATGGCGTTGACCTCAGCCCCCTGCTGTTGGGCACAGGCAAGAGCCCCCGGCACACCCTCTTCTTCTACTCGGCTTTCCCGGATGAGGTCCGAGGGGTCTTTGCTGTGCGGAGCGGGAAGTACAAGGCGCACTTCTTTACCCAGGGCTCTGTCCACAGCGACACCACTGCGGACCCTGCCTGCCACGCCTCTAGTCCTCTGACTGCCCATGAGCCCCCGCTGCTCTTTGACCTGTCTGAGGACCCTGGTGAGAACTACAACCTTCTGGAGGGTGTGGATAAGGTCGCCCCAGAGGCGCTGCAGGCAATGAAGCAACTTGAGCTGCTCAAGGCCCAGTTTGATGCTGCCATGACCTTTGGGCCCAGCCAGATGGCGCGGGGCGAGGACCCCGCCCTGCAGGTCTGCTGCCAGCCCAGCTGCACCCCCCGGCCGTCCTGCTGCCACTGCCCCGAGTTCCAGCCCTGAGGGCGCAGACGGAGGCCGTCTGGGGTTCCTGGCTGTGCTGTGGGGGTGTGGAGGTGGTTTGTACCCGAGAACTCTAATAACACCAGCTGACACTTGTGTGATAA

WT: GGTTCCTGGCTGTGCTGT

MU: TTTGAAGTTATTGTAGTG

Primer:

WT-F: CTAGCTAGCGGGGTTCCTGGCTGTGCTGT

MU-F: CTAGCTAGCGGTTTGAAGTTATTGTAGTG

R: CCGCTCGAGTTATCACACAAGTGTCAGCTGGTGT

Amplification product size:261bp 100bp

**3.2 pmirGLO Vector sequence**

CATGCAAGCTGATCCGGCTGCTAACAAAGCCCGAAAGGAAGCTGAGTTGGCTGCTGCCACCGCTGAGCAATAACTAGCATAACCCCTTGGGGCGGCCGCTTCGAGCAGACATGATAAGATACATTGATGAGTTTGGACAAACCACAACTAGAATGCAGTGAAAAAAATGCTTTATTTGTGAAATTTGTGATGCTATTGCTTTATTTGTAACCATTATAAGCTGCAATAAACAAGTTAACAACAACAATTGCATTCATTTTATGTTTCAGGTTCAGGGGGAGATGTGGGAGGTTTTTTTAAGCAAGTAAAACCTCTACAAATGTGGTAAAATCGAATTTTAACAAAATATTAACGCTTACAATTTCCTGATGCGGTATTTTCTCCTTACGCATCTGTGCGGTATTTCACACCGCATACGCGGATCTGCGCAGCACCATGGCCTGAAATAACCTCTGAAAGAGGAACTTGGTTAGGTACCTTCTGAGGCGGAAAGAACCAGCTGTGGAATGTGTGTCAGTTAGGGTGTGGAAAGTCCCCAGGCTCCCCAGCAGGCAGAAGTATGCAAAGCATGCATCTCAATTAGTCAGCAACCAGGTGTGGAAAGTCCCCAGGCTCCCCAGCAGGCAGAAGTATGCAAAGCATGCATCTCAATTAGTCAGCAACCATAGTCCCGCCCCTAACTCCGCCCATCCCGCCCCTAACTCCGCCCAGTTCCGCCCATTCTCCGCCCCATGGCTGACTAATTTTTTTTATTTATGCAGAGGCCGAGGCCGCCTCGGCCTCTGAGCTATTCCAGAAGTAGTGAGGAGGCTTTTTTGGAGGCCTAGGCTTTTGCAAAAAGCTTGATTCTTCTGACACAACAGTCTCGAACCAAAGGCTGGAGCCACCATGGCTTCCAAGGTGTACGACCCCGAGCAACGCAAACGCATGATCACTGGGCCTCAGTGGTGGGCTCGCTGCAAGCAAATGAACGTGCTGGACTCCTTCATCAACTACTATGATTCCGAGAAGCACGCCGAGAACGCCGTGATTTTTCTGCATGGTAACGCTGCCTCCAGCTACCTGTGGAGGCACGTCGTGCCTCACATCGAGCCCGTGGCTAGATGCATCATCCCTGATCTGATCGGAATGGGTAAGTCCGGCAAGAGCGGGAATGGCTCATATCGCCTCCTGGATCACTACAAGTACCTCACCGCTTGGTTCGAGCTGCTGAACCTTCCAAAGAAAATCATCTTTGTGGGCCACGACTGGGGGGCTTGTCTGGCCTTTCACTACTCCTACGAGCACCAAGACAAGATCAAGGCCATCGTCCATGCTGAGAGTGTCGTGGACGTGATCGAGTCCTGGGACGAGTGGCCTGACATCGAGGAGGATATCGCCCTGATCAAGAGCGAAGAGGGCGAGAAAATGGTGCTTGAGAATAACTTCTTCGTCGAGACCATGCTCCCAAGCAAGATCATGCGGAAACTGGAGCCTGAGGAGTTCGCTGCCTACCTGGAGCCATTCAAGGAGAAGGGCGAGGTTAGACGGCCTACCCTCTCCTGGCCTCGCGAGATCCCTCTCGTTAAGGGAGGCAAGCCCGACGTCGTCCAGATTGTCCGCAACTACAACGCCTACCTTCGGGCCAGCGACGATCTGCCTAAGATGTTCATCGAGTCCGACCCTGGGTTCTTTTCCAACGCTATTGTCGAGGGAGCTAAGAAGTTCCCTAACACCGAGTTCGTGAAGGTGAAGGGCCTCCACTTCAGCCAGGAGGACGCTCCAGATGAAATGGGTAAGTACATCAAGAGCTTCGTGGAGCGCGTGCTGAAGAACGAGCAGACCGGTGGTGGGAGCGGAGGTGGCGGATCAGGTGGCGGAGGCTCCGGAGGGATTGAACAAGATGGATTGCACGCAGGTTCTCCGGCCGCTTGGGTGGAGAGGCTATTCGGCTATGACTGGGCACAACAGACAATCGGCTGCTCTGATGCCGCCGTGTTCCGGCTGTCAGCGCAGGGGCGCCCGGTTCTTTTTGTCAAGACCGACCTGTCCGGTGCCCTGAATGAACTGCAGGACGAGGCAGCGCGGCTATCGTGGCTGGCCACGACGGGCGTTCCTTGCGCAGCTGTGCTCGACGTTGTCACTGAAGCGGGAAGGGACTGGCTGCTATTGGGCGAAGTGCCGGGGCAGGATCTCCTGTCATCTCACCTTGCTCCTGCCGAGAAAGTATCCATCATGGCTGATGCAATGCGGCGGCTGCATACGCTTGATCCGGCTACCTGCCCATTCGACCACCAAGCGAAACATCGCATCGAGCGAGCACGTACTCGGATGGAAGCCGGTCTTGTCGATCAGGATGATCTGGACGAAGAGCATCAGGGGCTCGCGCCAGCCGAACTGTTCGCCAGGCTCAAGGCGCGCATGCCCGACGGCGAGGATCTCGTCGTGACCCATGGCGATGCCTGCTTGCCGAATATCATGGTGGAAAATGGCCGCTTTTCTGGATTCATCGACTGTGGCCGGCTGGGTGTGGCGGACCGCTATCAGGACATAGCGTTGGCTACCCGTGATATTGCTGAAGAGCTTGGCGGCGAATGGGCTGACCGCTTCCTCGTGCTTTACGGTATCGCCGCTCCCGATTCGCAGCGCATCGCCTTCTATCGCCTTCTTGACGAGTTCTTCTGAGCGGGACTCTGGGGTTCGAAATGACCGACCAAGCGACGCCCAACCTGCCATCACGATGGCCGCAATAAAATATCTTTATTTTCATTACATCTGTGTGTTGGTTTTTTGTGTGAATCGATAGCGATAAGGATCCTCTTTGCGCTTGCGTTTTCCCTTGTCCAGATAGCCCAGTAGCTGACATTCATCCGGGGTCAGCACCGTTTCTGCGGACTGGCTTTCTACGTAATGGTTTCTTAGACGTCAGGTGGCACTTTTCGGGGAAATGTGCGCGGAACCCCTATTTGTTTATTTTTCTAAATACATTCAAATATGTATCCGCTCATGAGACAATAACCCTGATAAATGCTTCAATAATATTGAAAAAGGAAGAGTATGAGTATTCAACATTTCCGTGTCGCCCTTATTCCCTTTTTTGCGGCATTTTGCCTTCCTGTTTTTGCTCACCCAGAAACGCTGGTGAAAGTAAAAGATGCTGAAGATCAGTTGGGTGCACGAGTGGGTTACATCGAACTGGATCTCAACAGCGGTAAGATCCTTGAGAGTTTTCGCCCCGAAGAACGTTTTCCAATGATGAGCACTTTCAAAGTTCTGCTATGTGGCGCGGTATTATCCCGTATTGACGCCGGGCAAGAGCAACTCGGTCGCCGCATACACTATTCTCAGAATGACTTGGTTGAGTACTCACCAGTCACAGAAAAGCATCTTACGGATGGCATGACAGTAAGAGAATTATGCAGTGCTGCCATAACCATGAGTGATAACACTGCGGCCAACTTACTTCTGACAACTATCGGAGGACCGAAGGAGCTAACCGCTTTTTTGCACAACATGGGGGATCATGTAACTCGCCTTGATCGTTGGGAACCGGAGCTGAATGAAGCCATACCAAACGACGAGCGTGACACCACGATGCCTGTAGCAATGGCAACAACGTTGCGCAAACTATTAACTGGCGAACTACTTACTCTAGCTTCCCGGCAACAATTAATAGACTGGATGGAGGCGGATAAAGTTGCAGGACCACTTCTGCGCTCGGCCCTTCCGGCTGGCTGGTTTATTGCTGATAAATCTGGAGCCGGTGAGCGTGGGTCTCGCGGTATCATTGCAGCACTGGGGCCAGATGGTAAGCCCTCCCGTATCGTAGTTATCTACACGACGGGGAGTCAGGCAACTATGGATGAACGAAATAGACAGATCGCTGAGATAGGTGCCTCACTGATTAAGCATTGGTAATTCGAAATGACCGACCAAGCGACGCCCAACCGGTATCAGCTCACTCAAAGGCGGTAATACGGTTATCCACAGAATCAGGGGATAACGCAGGAAAGAACATGTGAGCAAAAGGCCAGCAAAAGGCCAGGAACCGTAAAAAGGCCGCGTTGCTGGCGTTTTTCCATAGGCTCCGCCCCCCTGACGAGCATCACAAAAATCGACGCTCAAGTCAGAGGTGGCGAAACCCGACAGGACTATAAAGATACCAGGCGTTTCCCCCTGGAAGCTCCCTCGTGCGCTCTCCTGTTCCGACCCTGCCGCTTACCGGATACCTGTCCGCCTTTCTCCCTTCGGGAAGCGTGGCGCTTTCTCATAGCTCACGCTGTAGGTATCTCAGTTCGGTGTAGGTCGTTCGCTCCAAGCTGGGCTGTGTGCACGAACCCCCCGTTCAGCCCGACCGCTGCGCCTTATCCGGTAACTATCGTCTTGAGTCCAACCCGGTAAGACACGACTTATCGCCACTGGCAGCAGCCACTGGTAACAGGATTAGCAGAGCGAGGTATGTAGGCGGTGCTACAGAGTTCTTGAAGTGGTGGCCTAACTACGGCTACACTAGAAGGACAGTATTTGGTATCTGCGCTCTGCTGAAGCCAGTTACCTTCGGAAAAAGAGTTGGTAGCTCTTGATCCGGCAAACAAACCACCGCTGGTAGCGGTGGTTTTTTTGTTTGCAAGCAGCAGATTACGCGCAGAAAAAAAGGATTTCAAGAAGATCCTTTGATCTTTTCTACGGGGTCTGACGCTCAGTGGAACGAAAACTCACGTTAAGGGATTTTGGTCATGAGATTATCAAAAAGGATCTTCACCTAGATCCTTTTATAGTCCGGAAATACAGGAACGCACGCTGGATGGCCCTTCGCTGGGATGGTGAAACCATGAAAAATGGCAGCTTCAGTGGATTAAGTGGGGGTAATGTGGCCTGTACCCTCTGGTTGCATAGGTATTCATACGGTTAAAATTTATCAGGCGCGATTGCGGCAGTTTTTCGGGTGGTTTGTTGCCATTTTTACCTGTCTGCTGCCGTGATCGCGCTGAACGCGTTTTAGCGGTGCGTACAATTAAGGGATTATGGTAAATCCACTTACTGTCTGCCCTCGTAGCCATCGAGATAAACCGCAGTACTCCGGCCACGATGCGTCCGGCGTAGAGGATCGAGATCTACCGGGTAGGGGAGGCGCTTTTCCCAAGGCAGTCTGGAGCATGCGCTTTAGCAGCCCCGCTGGGCACTTGGCGCTACACAAGTGGCCTCTGGCCTCGCACACATTCCACATCCACCGGTAGGCGCCAACCGGCTCCGTTCTTTGGTGGCCCCTTCGCGCCACCTTCTACTCCTCCCCTAGTCAGGAAGTTCCCCCCCGCCCCGCAGCTCGCGTCGTGCAGGACGTGACAAATGGAAGTAGCACGTCTCACTAGTCTCGTGCAGATGGACAGCACCGCTGAGCAATGGAAGCGGGTAGGCCTTTGGGGCAGCGGCCAATAGCAGCTTTGCTCCTTCGCTTTCTGGGCTCAGAGGCTGGGAAGGGGTGGGTCCGGGGGCGGGCTCAGGGGCGGGCTCAGGGGCGGGGCGGGCGCCCGAAGGTCCTCCGGAGGCCCGGCATTCTGCACGCTTCAAAAGCGCACGTCTGCCGCGCTGTTCTCCTCTTCCTCATCTCCGGGCCTTTCGACCTGCAGCCCAAGCTTGGCAATCCGGTACTGTTGGTAAAGCCACCATGGAAGATGCCAAAAACATTAAGAAGGGCCCAGCGCCATTCTACCCACTCGAAGACGGGACCGCCGGCGAGCAGCTGCACAAAGCCATGAAGCGCTACGCCCTGGTGCCCGGCACCATCGCCTTTACCGACGCACATATCGAGGTGGACATTACCTACGCCGAGTACTTCGAGATGAGCGTTCGGCTGGCAGAAGCTATGAAGCGCTATGGGCTGAATACAAACCATCGGATCGTGGTGTGCAGCGAGAATAGCTTGCAGTTCTTCATGCCCGTGTTGGGTGCCCTGTTCATCGGTGTGGCTGTGGCCCCAGCTAACGACATCTACAACGAGCGCGAGCTGCTGAACAGCATGGGCATCAGCCAGCCCACCGTCGTATTCGTGAGCAAGAAAGGGCTGCAAAAGATCCTCAACGTGCAAAAGAAGCTACCGATCATACAAAAGATCATCATCATGGATAGCAAGACCGACTACCAGGGCTTCCAAAGCATGTACACCTTCGTGACTTCCCATTTGCCACCCGGCTTCAACGAGTACGACTTCGTGCCCGAGAGCTTCGACCGGGACAAAACCATCGCCCTGATCATGAACAGTAGTGGCAGTACCGGATTGCCCAAGGGCGTAGCCCTACCGCACCGCACCGCTTGTGTCCGATTCAGTCATGCCCGCGACCCCATCTTCGGCAACCAGATCATCCCCGACACCGCTATCCTCAGCGTGGTGCCATTTCACCACGGCTTCGGCATGTTCACCACGCTGGGCTACTTGATCTGCGGCTTTCGGGTCGTGCTCATGTACCGCTTCGAGGAGGAGCTATTCTTGCGCAGCTTGCAAGACTATAAGATTCAATCTGCCCTGCTGGTGCCCACACTATTTAGCTTCTTCGCTAAGAGCACTCTCATCGACAAGTACGACCTAAGCAACTTGCACGAGATCGCCAGCGGCGGGGCGCCGCTCAGCAAGGAGGTAGGTGAGGCCGTGGCCAAACGCTTCCACCTACCAGGCATCCGCCAGGGCTACGGCCTGACAGAAACAACCAGCGCCATTCTGATCACCCCCGAAGGGGACGACAAGCCTGGCGCAGTAGGCAAGGTGGTGCCCTTCTTCGAGGCTAAGGTGGTGGACTTGGACACCGGTAAGACACTGGGTGTGAACCAGCGCGGCGAGCTGTGCGTCCGTGGCCCCATGATCATGAGCGGCTACGTTAACAACCCCGAGGCTACAAACGCTCTCATCGACAAGGACGGCTGGCTGCACAGCGGCGACATCGCCTACTGGGACGAGGACGAGCACTTCTTCATCGTGGACCGGCTGAAGAGCCTGATCAAATACAAGGGCTACCAGGTAGCCCCAGCCGAACTGGAGAGCATCCTGCTGCAACACCCCAACATCTTCGACGCCGGGGTCGCCGGCCTGCCCGACGACGATGCCGGCGAGCTGCCCGCCGCAGTCGTCGTGCTGGAACACGGTAAAACCATGACCGAGAAGGAGATCGTGGACTATGTGGCCAGCCAGGTTACAACCGCCAAGAAGCTGCGCGGTGGTGTTGTGTTCGTGGACGAGGTGCCTAAAGGACTGACCGGCAAGTTGGACGCCCGCAAGATCCGCGAGATTCTCATTAAGGCCAAGAAGGGCGGCAAGATCGCCGTGTAATTCTAGTTGTTTAAACGAGCTCGCTAGCCTCGAGTCTAGAGTCGACCTGCAGG


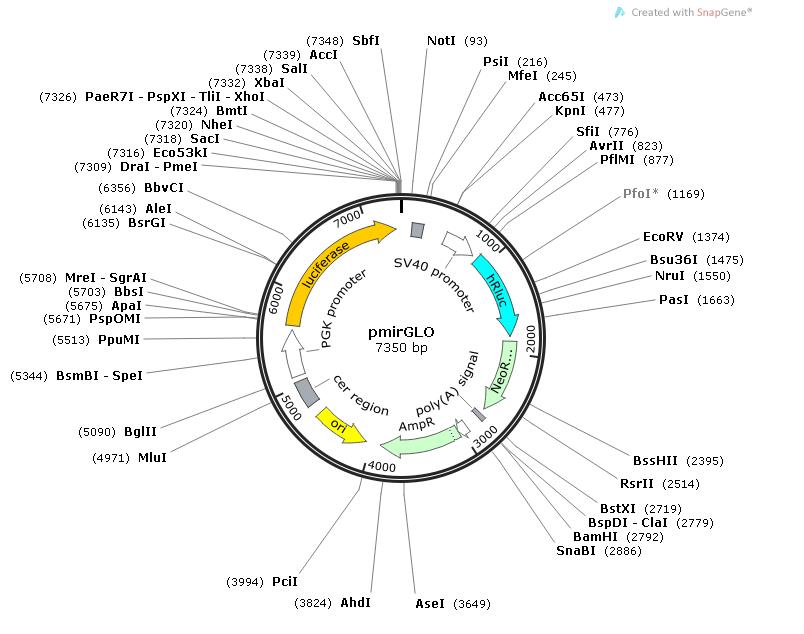


**4 PCR amplification**

1. PCR was conducted using PrimeSTAR^®^ HS DNA Polymerase:

| Composition | Volume |
| --- | --- |
| Template | 200ng |
| Forward | 2.5 μL(10μM) |
| Reverse | 2.5 μL(10μM) |
| 5×PrimeSTAR Buffer (Mg^2+^ plus) | 20 μL |
| dNTP | 5 μL |
| PrimeSTAR HS DNA Polymerase (2.5 U/μl) | 0.5 μL |
| Super pure water up to | 100 μL |

1. PCR conditions are as follows：98℃ 10s, 60℃ 15s, 72℃ 40s, 40 cycle，72℃ 5min,4℃ ∞。
2. The size of the target fragment was determined by 1.5% agarose at the end of PCR.
3. PCR amplification results


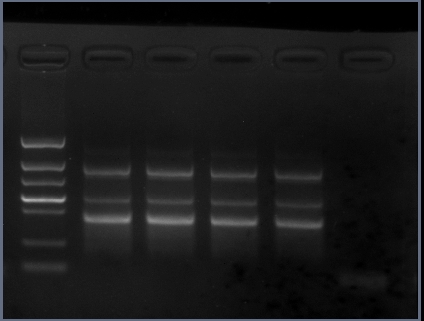


M 1 2 3 4 H_2_O

M:Marker;

1-4：01WT amplification

1500bp

500bp

200bp

100bp

400bp

700bp

900bp

400bp

100bp

200bp

500bp


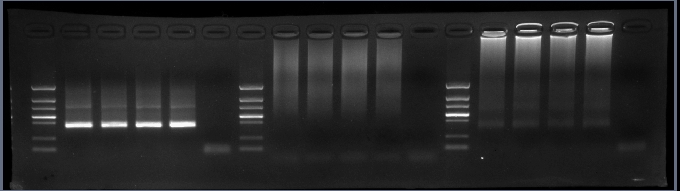


M:Marker;

1-4：01MU amplification

M 1 2 3 4 H_2_O

1500bp

1500bp

100bp

200bp

400bp

500bp

700bp

900bp


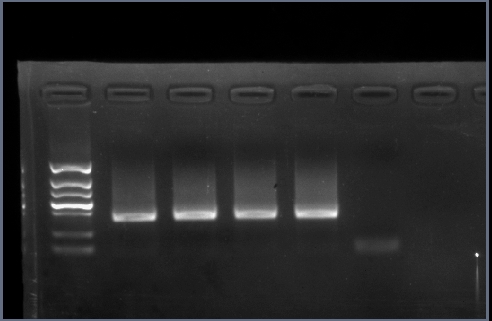


M 1 2 3 4 H_2_O

M:Marker;

1-4：39WT amplification


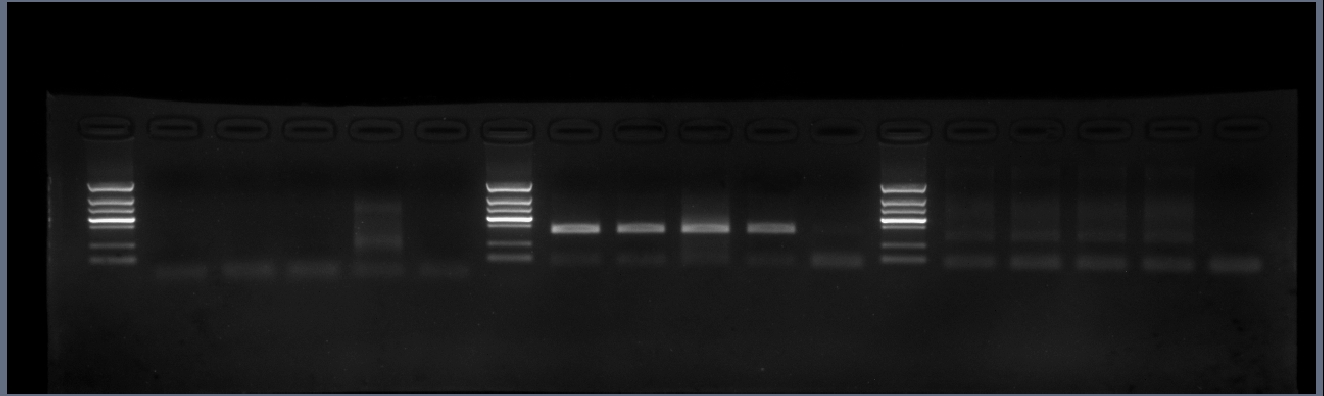


M 1 2 3 4 H_2_O

100bp

200bp

400bp

500bp

1500bp

M:Marker;

1-4：39MU amplification


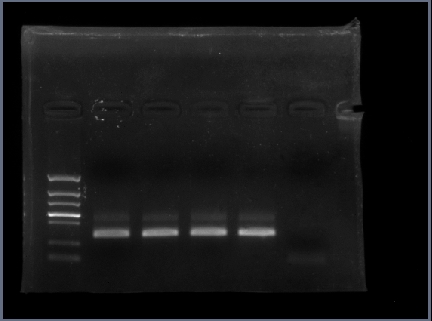


M 1 2 3 4 H_2_O

100bp

200bp

400bp

500bp

1500bp

M:Marker;

1-4：41WT amplification


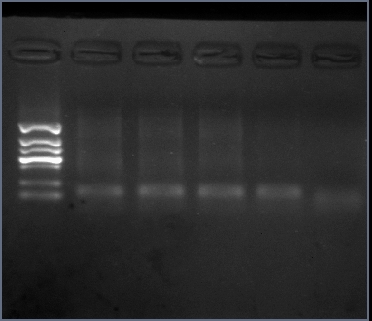


M:Marker;

1-4：96WT amplification

1500bp

100bp

200bp

400bp

500bp

M 1 2 3 4 H_2_O


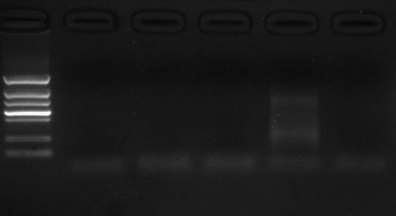


M 1 2 3 4 H_2_O

M:Marker;

1-4：96MU amplification

No target bands were amplified.


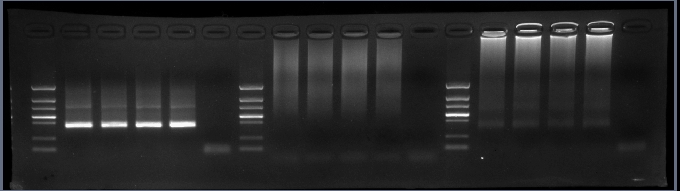


M 1 2 3 4 H_2_O

M:Marker;

1-4：41MU amplification

The target band is diffuse and weak.

**5 First round PCR**

XM_018063741.1-MU, XM_005701596.3-MU

No target bands were amplified. The gene synthesis was done. The amplification bands were all in line with the target band size and were cut and recycled.


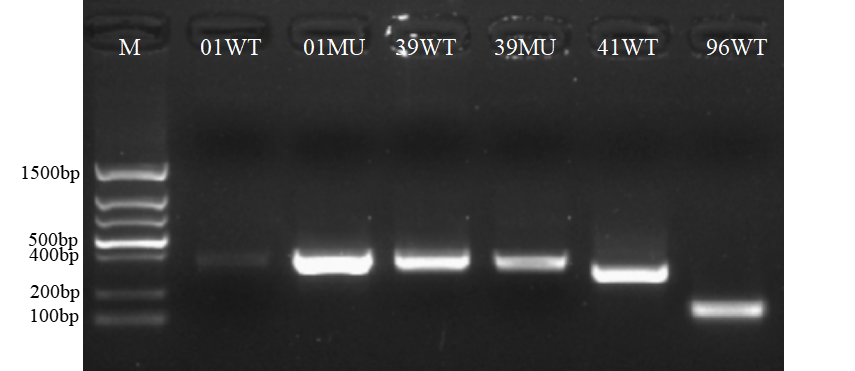


The product and target vector pmirGLO were digested overnight with double enzyme digestion.

**6 Double enzyme digestion**

| Composition | Volume |
| --- | --- |
| PCR Recycled product/pmirGLO | 1 μg |
| 10×rCutSmart Buffer | 5 μL |
| NheI-HF | 1 μL |
| XhoI | 1 μL |
| H_2_O up to | 50 μL |

Reaction at 37℃ overnight, glue cutting recovery.

M:Marker;

1：pmirGLO Double enzyme digestion recovery.


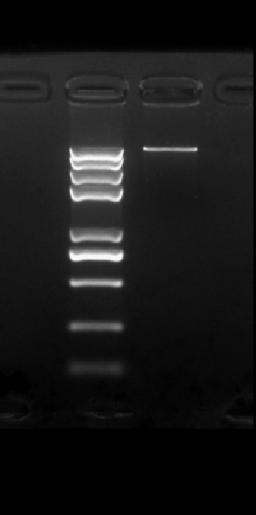


M 1

750bp

2000bp

8000bp


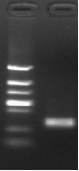

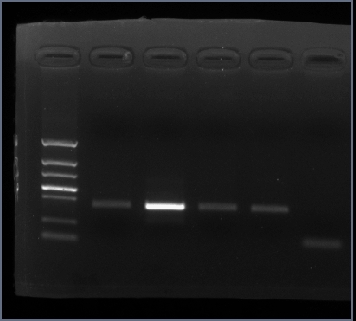


M 01WT 01MU39WT 39MU96WT

1500bp

200bp

100bp

400bp

500bp

M 41WT

**7** **Ligase**

| Composition | Volume |
| --- | --- |
| Target fragment after Double enzyme digestion (<20ng/μL) | 7μL |
| Double enzyme digestion recovery pmirGLO（20ng/μL） | 1 μL |
| T4 DNA ligase | 1 μL |
| 10×T4 Buffer | 1 μL |

16℃ connection reaction overnight.

**8** **Transform**

1. 10μL of the connecting product was added to 50ul of escherichia coli induced state, and stood on ice for 30min.
2. 42℃ hot hit 90 seconds, quickly remove and place on ice.
3. Add 1mL LB medium and resume culture for 1 hour.
4. Spread the recovered culture solution on LB/Amp/(100μg/mL) AGAR plate, dry, and culture overnight at 37℃.
5. Single colonies were selected on the next day and inoculated in 5mL LB/Amp(100μg/mL) liquid medium at 220rpm at 37℃.

**9 Colony PCR validation and sequencing**

Single colony was selected for PCR amplification verification. PCR amplification procedure: 98℃ 10s, 60℃ 15s, 72℃ 40s, 30 cycle，72℃ 5min,4℃ ∞. Correct clones were selected for bacterial liquid sequencing verification. The sequencing company was Genewiz, China, and the sequencing primer was pmirGLO-F（01WT：14/16；01MU：10；39WT：5/10；39MU：5/24；41WT：8/18；96WT：10/23）。


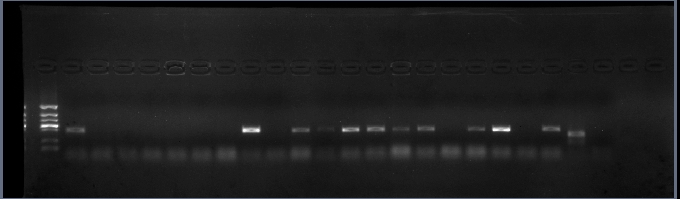


M 1 2 3 4 5 6 7 8 9 10 11 12 13 14 15 16 17 18 19 20 阳 阴

41WT


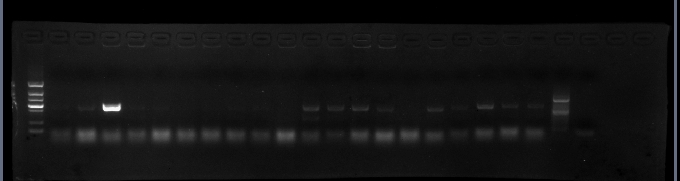

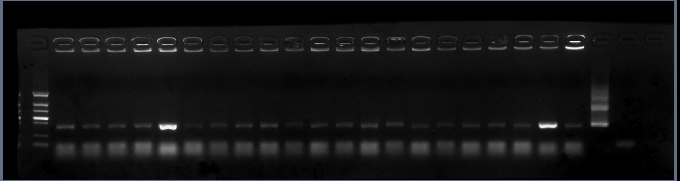


M 1 2 3 4 5 6 7 8 9 10 11 12 13 14 15 16 17 18 19 20 21 阳 阴


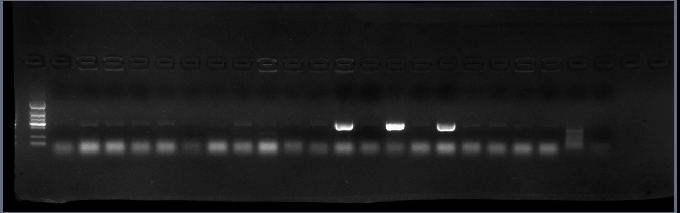


M 1 2 3 4 5 6 7 8 9 10 11 12 13 14 15 16 17 18 19 20 阳 阴

01WT


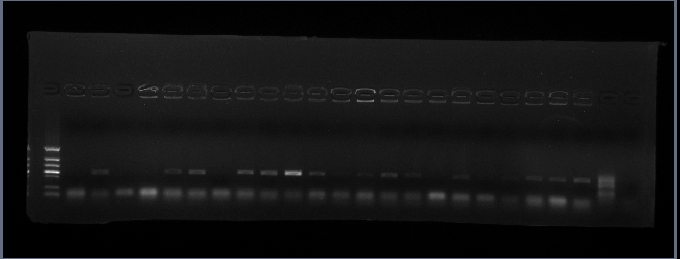


M 1 2 3 4 5 6 7 8 9 10 11 12 13 14 15 16 17 1819 20 21 22 阳 阴

01MU


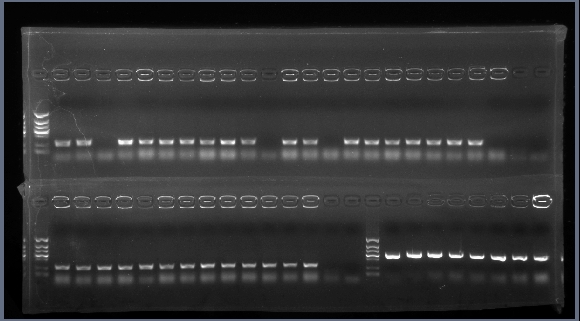

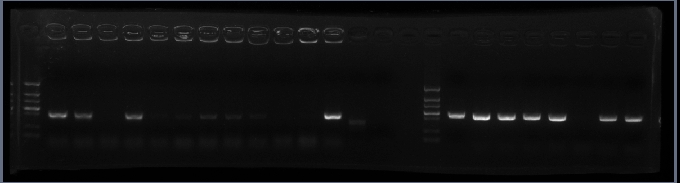


M 1 2 3 4 5 6 7 8 M 9 10 11 12 13 14 15 16

39WT


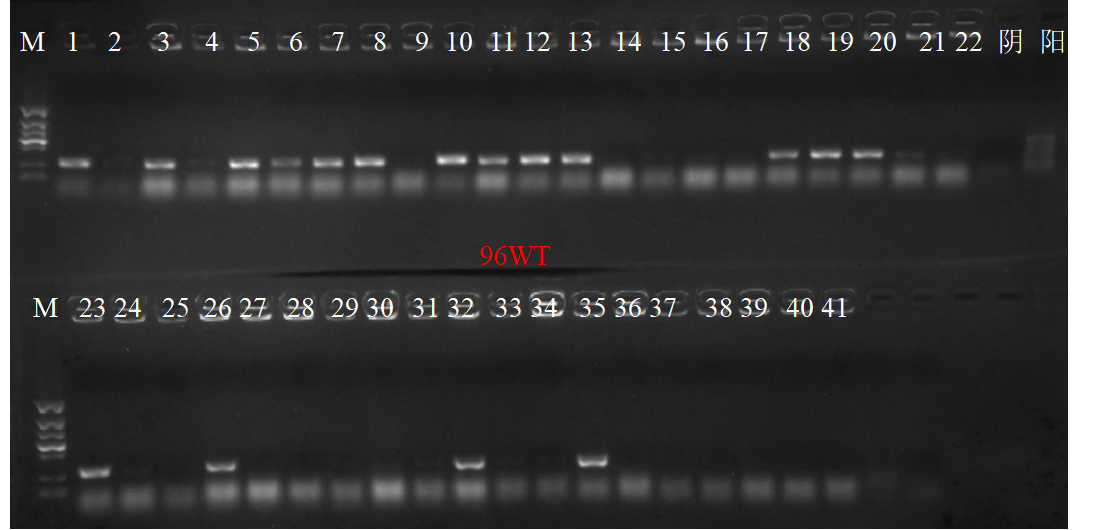

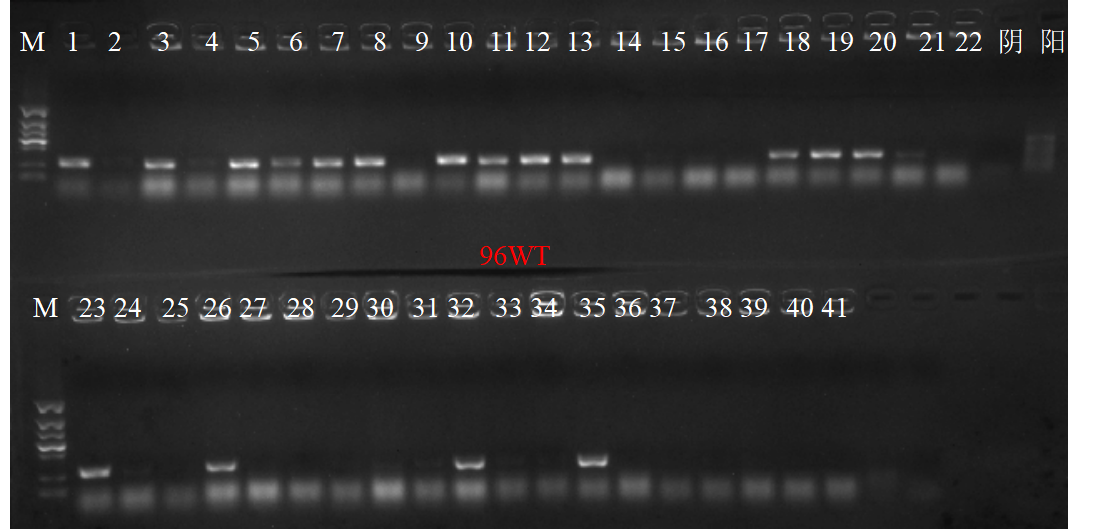


**10 Sequence results**

Blue is the vector sequence, yellow is the restriction site, yellow is the target fragment, and red is the action site.

**10.1 XM_005676701.3-WT**

GGGCCACTTCAGGCCCTTCCGTGTGTTGTCTGGCGGGGTACCTTGTCCAGCCAGCCACCCACTTTGACTCCCCCGTAGCTTAGGACACAAGCCAGCTACCAGCGGTACAGAGCAGTGATCAAAGCCGAGTACTTACAACTCTGGTAAGCCCAACTTCTCCGCCTCAACCCTTCTGCCTCTTGGAGGGATACGCTGGGGGTGAGCTGCTTGAGATTCTCGACAGGCTTCTGCAAAAGCTCTTCCCTCCTGAAGGCAGATCCAGTCTTGGTGGCTCTCACCCTCCACGCTGGTAAAGCTGCACCTCTCTTGGGGGGACGAGGGGCTGCAGGAATCCCTGGAGACCCTGGTGCTTCACGATGCTGCTCCGGTGATTCTTGTACATAATCTGGTGTGTTCACCA

10.1.1 >Pmir-01WT-14-PmirGLO-F_A10

CCCGGATGGCGCCGCAAGATCCGCGAGATTCTCATTAAGGCCAAGAAGGGCGGCAAGATCGCCGTGTAATTCTAGTTGTTTAAACGAGCTCGCTAGCTACCTTGTCCAGCCAGCCACCCACTTTGACTCCCCCGTAGCTTAGGACACAAGCCAGCTACCAGCGGTACAGAGCAGTGATCAAAGCCGAGTACTTACAACTCTGGTAAGCCCAACTTCTCCGCCTCAACCCTTCTGCCTCTTGGAGGGATACGCTGGGGGTGAGCTGCTTGAGATTCTCGACAGGCTTCTGCAAAAGCTCTTCCCTCCTGAAGGCAGATCCAGTCTTGGTGGCTCTCACCCTCCACGCTGGTAAAGCTGCACCTCTCTTGGGGGGACGAGGGGCTGCAGGAATCCCTGGAGACCCTGGTGCTTCACGATGCTGCTCTCGAGTCTAGAGTCGACCTGCAGGCATGCAAGCTGATCCGGCTGCTAACAAAGCCCGAAAGGAAGCTGAGTTGGCTGCTGCCACCGCTGAGCAATAACTAGCATAACCCCTTGGGGCGGCCGCTTCGAGCAGACATGATAAGATACATTGATGAGTTTGGACAAACCACAACTAGAATGCAGTGAAAAAAATGCTTTATTTGTGAAATTTGTGATGCTATTGCTTTATTTGTAACCATTATAAGCTGCAATAAACAAGTTAACAACAACAATTGCATTCATTTTATGTTTCAGGTTCAGGGGGAGATGTGGGAGGTTTTTTTAAGCAAGTAAAACCTCTACAAATGTGGTAAAATCGA

ATTTTAACAAAATATTAACGCTTACAATTTCCTGATGCGGTATTTTCTCCTTACGCATCTGTGCGGTATTTCACACCGCATACGCGGATCTGCGCAGCACCATGGCCTGAAATAACCTCTGAAAGAGGAACTTGGTTAGGTACCTTCTGAGGCGGAAAGAACCAGCTGTGGAATGTGTGTCAGTTAAGGGTGTGGAAAGGCCCCAGGCTCCCCAGCAGGCAGAGTTTGCAAAGCTTGCTTTCATTAGTCAGAACCAGGGGG


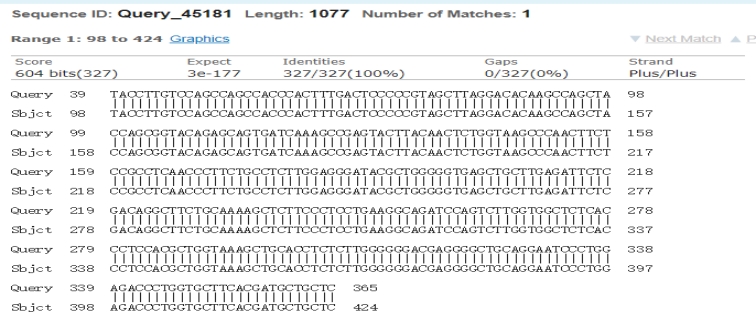
GAAAGCCCCAGGTTCCCACAGGGAAATTTGCAAA

Correct

10.1.2 >Pmir-01WT-16-PmirGLO-F_B10

TCCGGATGGCGCCGCAAGATCCGCGAGATTCTCATTAAGGCCAAGAAGGGCGGCAAGATCGCCGTGTAATTCTAGTTGTTTAAACGAGCTCGCTAGCTACCTTGTCCAGCCAGCCACCCACTTTGACTCCCCCGTAGCTTAGGACACAAGCCAGCTACCAGCGGTACAGAGCAGTGATCAAAGCCGAGTACTTACAACTCTGGTAAGCCCAACTTCTCCGCCTCAACCCTTCTGCCTCTTGGAGGGATACGCTGGGGGTGAGCTGCTTGAGATTCTCGACAGGCTTCTGCAAAAGCTCTTCCCTCCTGAAGGCAGATCCAGTCTTGGTGGCTCTCACCCTCCACGCTGGTAAAGCTGCACCTCTCTTGGGGGGACGAGGGGCTGCAGGAATCCCTGGAGACCCTGGTGCTTCACGATGCTGCTCTCGAGTCTAGAGTCGACCTGCAGGCATGCAAGCTGATCCGGCTGCTAACAAAGCCCGAAAGGAAGCTGAGTTGGCTGCTGCCACCGCTGAGCAATAACTAGCATAACCCCTTGGGGCGGCCGCTTCGAGCAGACATGATAAGATACATTGATGAGTTTGGACAAACCACAACTAGAATGCAGTGAAAAAAATGCTTTATTTGTGAAATTTGTGATGCTATTGCTTTATTTGTAACCATTATAAGCTGCAATAAACAAGTTAACAACAACAATTGCATTCATTTTATGTTTCAGGTTCAGGGGGAGATGTGGGGAGGTTTTTTTAAGCAAGTAAAACCTCTACAAATGTGGTAAAATCG

AATTTTAACAAAATATTAACGCTTACAATTTCCTGATGCGGTATTTTCTCCTTACGCATCTGTGCGGTATTTCACACCGCATACGCGGATCTGCGCAGCACCATGGGCCTGAAATAACCTCTGAAAGAGGAACTTGGTTAGGTACCTTCTGAGGCGGAAAGAACCAGCTGTGGAAAGGGTGTCAATTAAGGGTGTGGAAAAGCCCCCGGCTCCCCCGCAGGGGAAAATTTGCAAAGCTTGCTTTCCATTAGTCCGCACCCGGG


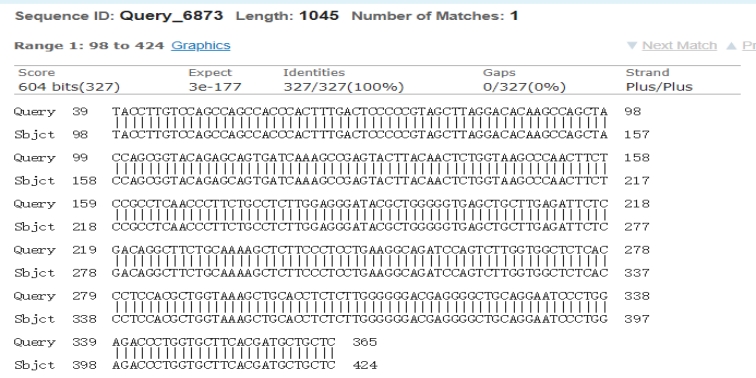


Correct

**10.2 XM_005676701.3-MU**

GGGCCACTTCAGGCCCTTCCGTGTGTTGTCTGGCGGGGTACCTTGTCCAGCCAGCCACCCACTTTGACTCCCCCGTAGCTTAGGACACAAGCCAGCTACCAGCGGTACAGAGCAGTGATCAAAGCCGAGTACTTACAACTCTGGTAAGCCCAACTTCTCCGCCTCAACCCTTCTGCCTCTTGGAGGGATACGCTGGGGGTGAGCTGCTTGAGATTCTCGACAGGCTTCTGCAAAAGCTCTTCCCTCCTGAAGGCAGATCCAGTCTTGGTGGCTCTCACCCTCCACGCTGGTAAAGCTGCACCTCTCTTGGGGGGACGAGGGGCTGCAGGAATCCCTGGAGACCCTGGTGCTTCCATCGTAGTAGCCGGTGATTCTTGTACATAATCTGGTGTGTTCACCA

10.2.1 >01Mu-10-pmirGLO-F_E12

CCCGGATGGAGCCGCAAGATCCGCGAGATTCTCATTAAGGCCAAGAAGGGCGGCAAGATCGCCGTGTAATTCTAGTTGTTTAAACGAGCTCGCTAGCTACCTTGTCCAGCCAGCCACCCACTTTGACTCCCCCGTAGCTTAGGACACAAGCCAGCTACCAGCGGTACAGAGCAGTGATCAAAGCCGAGTACTTACAACTCTGGTAAGCCCAACTTCTCCGCCTCAACCCTTCTGCCTCTTGGAGGGATACGCTGGGGGTGAGCTGCTTGAGATTCTCGACAGGCTTCTGCAAAAGCTCTTCCCTCCTGAAGGCAGATCCAGTCTTGGTGGCTCTCACCCTCCACGCTGGTAAAGCTGCACCTCTCTTGGGGGGACGAGGGGCTGCAGGAATCCCTGGAGACCCTGGTGCTTCCATCGTAGTAGCTCGAGTCTAGAGTCGACCTGCAGGCATGCAAGCTGATCCGGCTGCTAACAAAGCCCGAAAGGAAGCTGAGTTGGCTGCTGCCACCGCTGAGCAATAACTAGCATAACCCCTTGGGGCGGCCGCTTCGAGCAGACATGATAAGATACATTGATGAGTTTGGACAAACCACAACTAGAATGCAGTGAAAAAAATGCTTTATTTGTGAAATTTGTGATGCTATTGCTTTATTTGTAACCATTATAAGCTGCAATAAACAAGTTAACAACAACAATTGCATTCATTTTATGTTTCAGGTTCAGGGGGAGATGTGGGGAAGGTTTTTTTAAGCAAGTAAAACCTCTACAAATGTGGTAAAATCGAATTTTAACAAAATATTAACGCTTACAATTTCCTGATGCGGTATTTTCTCCTTACGCATCTGTGCGGTATTTCACACCGCATACGCGGATCTGCGCAGCACCATGGCCTGAAATAACCTCTGAAAGAGGAACTTGGTTAGGTACCTTCTGAGGCGGAAAGAACCAGCTGTGGAATGTGTGTCAGTTAGGGGTGTGGAAAGTCCCCAGGC


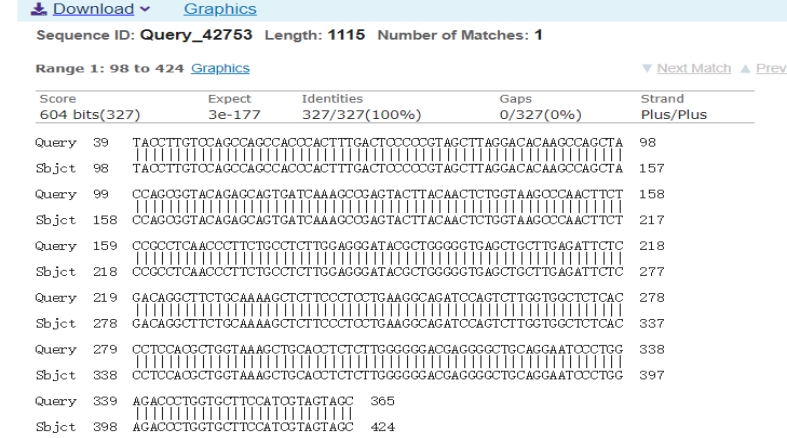
TCCCCAGCAGGCAGAAGTATGCAAAGCATGCATCTCAATTAGTCAGCAACCAGGTGTGGAAAGTCCCCAGGCTCCCCAGCAGGCAGAAGTATGCAAAGCATGCATCTCATTAGCCCGCCACCC

Correct

**10.3 XM_018064739.1-WT**

GGCACTAGAACTGGGTTGGGGGGGGGCCCTGTCCCTGATCCTGGGACCAGAAGACAGCTCCAGGAGGCAGCCCTCCTTTCTCACCACTCTCTGTCCTGTCCTGAGCTCGGCTTGTGACGGTCCGTTGTGATGTCCACACATGGTAAATCCTCGGGCTCCTGGAAGGCAACTGCCAGTTTGGTGCAAGAATCATCTGTCATTCGTAGACTTTGTGCGTCTTACAAAGTCACTGGCCTGTTTCACTGCTATCGTCATGGTACTAAGACGATACCCACCCTTGATCAGAGGCTGCACATGGCAAGTCAGTGGCTCTTGAGGGACAAATCTTCGTTTCCTGCTTCTGTACCACAAGGCGAGTAAGCATCCCCTTGGCCTCCCTTGTGGCTCAGCTGGTAAAGAATCCGCCTGCAATGTGGGAGACCTGGGTTGGGAAGATCCCCTGGAGAAGGGAATGGCTACCCACTCCAGTATTCTGGCCTG

10.3.1 >39WT-5-pmirGlo-F_A12

CCCGATGGAGCCGCAAGATCCGCGAGATTCTCATTAAGGCCAAGAAGGGCGGCAAGATCGCCGTGTAATTCTAGTTGTTTAAACGAGCTCGCTAGCAGCCCTCCTTTCTCACCACTCTCTGTCCTGTCCTGAGCTCGGCTTGTGACGGTCCGTTGTGATGTCCACACATGGTAAATCCTCGGGCTCCTGGAAGGCAACTGCCAGTTTGGTGCAAGAATCATCTGTCATTCGTAGACTTTGTGCGTCTTACAAAGTCACTGGCCTGTTTCACTGCTATCGTCATGGTACTAAGACGATACCCACCCTTGATCAGAGGCTGCACATGGCAAGTCAGTGGCTCTTGAGGGACAAATCTTCGTTTCCTGCTTCTGTACCACAAGGCGAGTAAGCATCCCCTTGGCCTCCCTTGTCTCGAGTCTAGAGTCGACCTGCAGGCATGCAAGCTGATCCGGCTGCTAACAAAGCCCGAAAGGAAGCTGAGTTGGCTGCTGCCACCGCTGAGCAATAACTAGCATAACCCCTTGGGGCGGCCGCTTCGAGCAGACATGATAAGATACATTGATGAGTTTGGACAAACCACAACTAGAATGCAGTGAAAAAAATGCTTTATTTGTGAAATTTGTGATGCTATTGCTTTATTTGTAACCATTATAAGCTGCAATAAACAAGTTAACAACAACAATTGCATTCATTTTATGTTTCAGGTTCAGGGGGAGATGTGGGAGGTTTTTTTAAGCAAGTAAAACCTCTACAAATGTGGTAAAATCGAATTTTAACAAAATATTAACGCTTACAATTTCCTGATGCGGTATTTTCTCCTTACGCATCTGTGCGGTATTTCACACCGCATACGCGGATCTGCGCAGCACCATGGCCTGAAATAACCTCTGAAAGAGGAACTTGGTTAGGTACCTTCTGAGGCGG


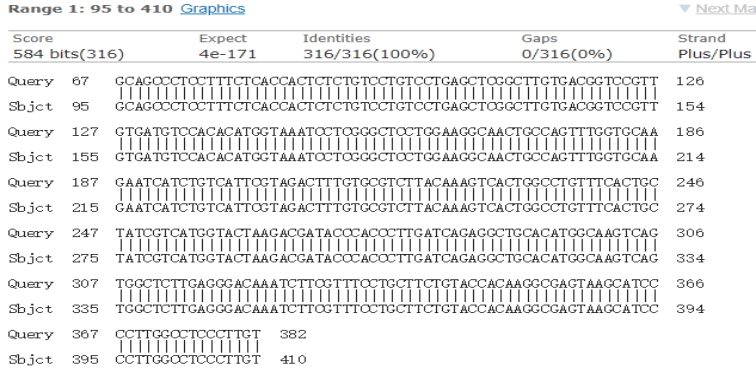


Correct

10.3.2 >39WT-10-pmirGlo-F_B12

CCCCGGATGGAGCCGCAAGATCCGCGAGATTCTCATTAAGGCCAAGAAGGGCGGCAAGATCGCCGTGTAATTCTAGTTGTTTAAACGAGCTCGCTAGCAGCCCTCCTTTCTCACCACTCTCTGTCCTGTCCTGAGCTCGGCTTGTGACGGTCCGTTGTGATGTCCACACATGGTAAATCCTCGGGCTCCTGGAAGGCAACTGCCAGTTTGGTGCAAGAATCATCTGTCATTCGTAGACTTTGTGCGTCTTACAAAGTCACTGGCCTGTTTCACTGCTATCGTCATGGTACTAAGACGATACCCACCCTTGATCAGAGGCTGCACATGGCAAGTCAGTGGCTCTTGAGGGACAAATCTTCGTTTCCTGCTTCTGTACCACAAGGCGAGTAAGCATCCCCTTGGCCTCCCTTGTCTCGAGTCTAGAGTCGACCTGCAGGCATGCAAGCTGATCCGGCTGCTAACAAAGCCCGAAAGGAAGCTGAGTTGGCTGCTGCCACCGCTGAGCAATAACTAGCATAACCCCTTGGGGCGGCCGCTTCGAGCAGACATGATAAGATACATTGATGAGTTTGGACAAACCACAACTAGAATGCAGTGAAAAAAATGCTTTATTTGTGAAATTTGTGATGCTATTGCTTTATTTGTAACCATTATAAGCTGCAATAAACAAGTTAACAACAACAATTGCATTCATTTTATGTTTCAGGTTCAGGGGGAGATGTGGGAGGTTTTTTTAAGCAAGTAAAACCTCTACAAATGTGGTAAAATCGAATTTTAACAAAATATTAACGCTTACAATTTCCTGATGCGGTATTTTCTCCTTACGCATCTGTGCGGTATTTCACACCGCATACGCGGATCTGCGCAGCACCATGGCCTGAAATAACCTCTGAAAGAGGAACTTGGTTAGGTACCTTCTGAGGCGGAAGGA


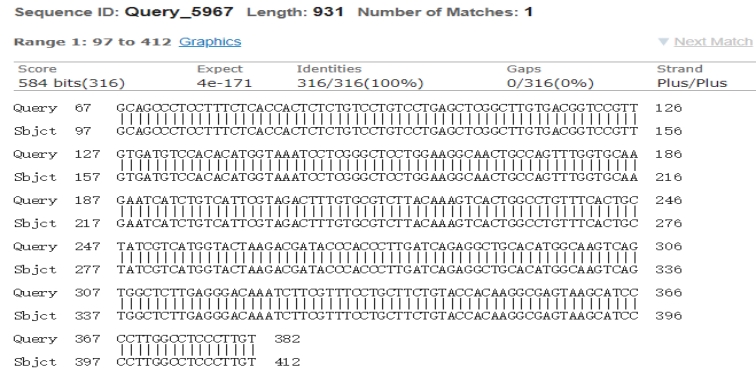


Correct

**10.4 XM_018064739.1-MU**

GGCACTAGAACTGGGTTGGGGGGGGGCCCTGTCCCTGATCCTGGGACCAGAAGACAGCTCCAGGAGGCAGCCCTCCTTTCTCACCACTCTCTGTCCTGTCCTGAGCTCGGCTTGTGACGGTCCGTTGTGATGTCCACACATGGTAAATCCTCGGGCTCCTGGAAGGCAACTGCCAGTTTGGTGCAAGAATCATCTGTCATTCGTAGACTTTGTGCGTCTTACAAAGTCACTGGCCTGTTTCACTGCTATCGTCATGGTACTAAGACGATACCCACCCTTGATCAGAGGCTGCACATGGCAAGTCAGTGGCTCTTGAGGGACAAATCTTCGTTTCCTGCTTCTGTACCACAAGGCGAGTAAGCATCCCCTTGTAAGAAATTGTGGCTCAGCTGGTAAAGAATCCGCCTGCAATGTGGGAGACCTGGGTTGGGAAGATCCCCTGGAGAAGGGAATGGCTACCCACTCCAGTATTCTGGCCTG

10.4.1 >Pmir-39MU-5-PmirGLO-F_E09

CCCGATTGGAGCCGCAAGATCCGCGAGATTCTCATTAAGGCCAAGAAGGGCGGCAAGATCGCCGTGTAATTCTAGTTGTTTAAACGAGCTCGCTAGCAGCCCTCCTTTCTCACCACTCTCTGTCCTGTCCTGAGCTCGGCTTGTGACGGTCCGTTGTGATGTCCACACATGGTAAATCCTCGGGCTCCTGGAAGGCAACTGCCAGTTTGGTGCAAGAATCATCTGTCATTCGTAGACTTTGTGCGTCTTACAAAGTCACTGGCCTGTTTCACTGCTATCGTCATGGTACTAAGACGATACCCACCCTTGATCAGAGGCTGAACATGGCAAGTCAGTGGCTCTTGAGGGACAAATCTTCGTTTCCTGCTTCTGTACCACAAGGCGAGTAAGCATCCCCTTGTAAGAAACTCGAGTCTAGAGTCGACCTGCAGGCATGCAAGCTGATCCGGCTGCTAACAAAGCCCGAAAGGA

AGCTGAGTTGGCTGCTGCCACCGCTGAGCAATAACTAGCATAACCCCTTGGGGCGGCCGCTTCGAGCAGACATGATAAGATACATTGATGAGTTTGGACAAACCACAACTAGAATGCAGTGAAAAAAATGCTTTATTTGTGAAATTTGTGATGCTATTGCTTTATTTGTAACCATTATAAGCTGCAATAAACAAGTTAACAACAACAATTGCATTCATTTTATGTTTCAGGTTCAGGGGGAGATGTGGGGAGGTTTTTTTAAGCAAGTAAAACCTCTACAAATGTGGTAAAATCGAATTTTAACAAAATATTAACGCTTACAATTTCCTGATGCGGTATTTTCTCCTTACGCATCTGTGCGGTATTTCACACCGCATACGCGGATCTGCGCAGCACCATGGCCTGAAATAACCTCTGAAAGAGGAACTTGGTTAGGTACCTTCTGAGGCGGAAAGAACCAGCTGTGGAATGGGTGTCAGTTAAGGGTGTGGAAAGGCCCCAGGCTCCCCAGCAGGCAGAGTTTG

CAAAGCTTGCTTTCCATTAGTCACCACCCGGGGTGGAAAGCCCCAGCTCCCCGCAGGGAAAATTTGCAAGCTGTTCTTTTTTTGCGCCCCATTTC


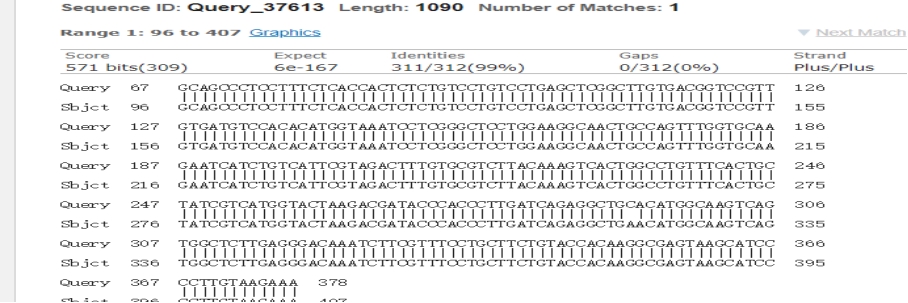


One base is mutated.

10.4.2 >pmir-39MU-24-pmirGLO-F_F06

TACGGATGGAGCCGCAAGATCCGCGAGATTCTCATTAAGGCCAAGAAGGGCGGCAAGATCGCCGTGTAATTCTAGTTGTTTAAACGAGCTCGCTAGCAGCCCTCCTTTCTCACCACTCTCTGTCCTGTCCTGAGCTCGGCTTGTGACGGTCCGTTGTGATGTCCACACATGGTAAATCCTCGGGCTCCTGGAAGGCAACTGCCAGTTTGGTGCAAGAATCATCTGTCATTCGTAGACTTTGTGCGTCTTACAAAGTCACTGGCCTGTTTCACTGCTATCGTCATGGTACTAAGACGATACCCACCCTTGATCAGAGGCTGCACATGGCAAGTCAGTGGCTCTTGAGGGACAAATCTTCGTTTCCTGCTTCTGTACCACAAGGCGAGTAAGCATCCCCTTGTAAGAAACTCGAGTCTAGAGTCGACCTGCAGGCATGCAAGCTGATCCGGCTGCTAACAAAGCCCGAAAGGAAGCTGAGTTGGCTGCTGCCACCGCTGAGCAATAACTAGCATAACCCCTTGGGGCGGCCGCTTCGAGCAGACATGATAAGATACATTGATGAGTTTGGACAAACCACAACTAGAATGCAGTGAAAAAAATGCTTTATTTGTGAAATTTGTGATGCTATTGCTTTATTTGTAACCATTATAAGCTGCAATAAACAAGTTAACAACAACAATTGCATTCATTTTATGTTTCAGGTTCAGGGGGAGATGTGGGAGGTTTTTTTAAGCAAGTAAAACCTCTACAAATGTGGTAAAATCGAATTTTAACAAAATATTAACGCTTACAATTTCCTGATGCGGTATTTTCTCCTTACGCATCTGTGCGGTATTTCACACCGCATACGCGGATCTGCGCAGCACCATGGCCTGAAATAACCTCTGAAAGAGGAACTTGGTTAGGTACCTTCTGAGGCGGAAAGAACCAGCTGTGGAATGTGTGTCAGTTAGGGTGTGGAAAGTCCCAGGCTCCCCAGCAGGCAGAATATGCAAAGCATGCTTTCATTAGTCACAACCAGGGGGGAAAGCCCCAGGTCCCCACAGG

GAAAATTTGAAGATGATCATTATTTAGACAGAAAAAAACGCGCGCCCAAACCCGCCCTCCGCCCAAAATCCCGCCTCGCCCCTTTCCGCCCGGGGAAAATTTTTTTTTTAAAAAGAGGCGCC


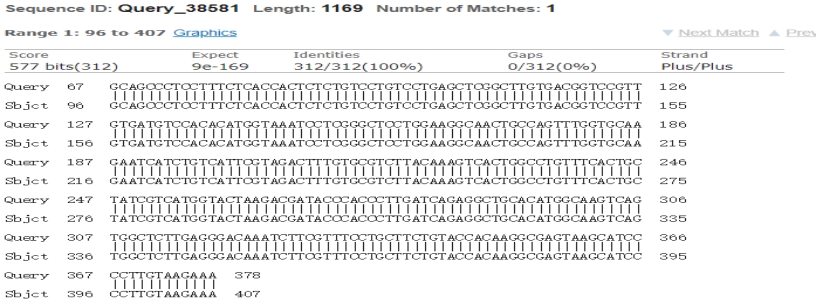


Correct

**10.5 XM_018063741.1-WT**

GGGGACTGTGTCTGGACCGAGCTGTGCACTTGCACAGCACCCTGCCCACACCCCAGAGACCCCATGGGGCTGGCCCTGCTCCCAGGAGCTCCCTCCCCCAGGAGAGGCCAAGTCCGAGGGGAGACTCTCCTAAAGGTGGGCTTCTTGTGGGGCCGAGGCCCCCTTCCTCAGGCCTGGGCTTGGGGACACTCGGAGCCTGTACATCTCTGCAGCTCGGGGCCCAGGCCCCGACAGACCTCCACTGCCCGGCCGTCGGGACCCCTCCTGGTCCTGGGATGCTGCTCAGCCCCTACCTCCGGC

10.5.1 >Pmir-41WT-8-PmirGLO-F_G09

TCCGATGGCGCCGCAAGATCCGCGAGATTCTCATTAAGGCCAAGAAGGGCGGCAAGATCGCCGTGTAATTCTAGTTGTTTAAACGAGCTCGCTAGCCCTGCCCACACCCCAGAGACCCCATGGGGCTGGCCCTGCTCCCAGGAGCTCCCTCCCCCAGGAGAGGCCAAGTCCGAGGGGAGACTCTCCTAAAGGTGGGCTTCTTGTGGGGCCGAGGCCCCCTTCCTCAGGCCTGGGCTTGGGGACACTCGGAGCCTGTACATCTCTGCAGCTCGGGGCCCAGGCCCCGACAGACCTCCACTGCCCGGCCGTCGGGACCCCTCCTGGTCCTGGGATGCTGCTCAGCCCTCGAGTCTAGAGTCGACCTGCAGGCATGCAAGCTGATCCGGCTGCTAACAAAGCCCGAAAGGAAGCTGAGTTGGCTGCTGCCACCGCTGAGCAATAACTAGCATAACCCCTTGGGGCGGCCGCTTCGAGCAGACATGATAAGATACATTGATGAGTTTGGACAAACCACAACT

AGAATGCAGTGAAAAAAATGCTTTATTTGTGAAATTTGTGATGCTATTGCTTTATTTGTAACCATTATAAGCTGCAATAAACAAGTTAACAACAACAATTGCATTCATTTTATGTTTCAGGTTCAGGGGGAGATGTGGGGAGGTTTTTTTAAGCAAGTAAAACCTCTACAAATGTGGTAAAATCGAATTTTAACAAAATATTAACGCTTACAATTTCCTGATGCGGTATTTTCTCCTTACGCATCTGTGCGGTATTTCACACCGCATACGCGGATCTGCGCAGCACCATGGCCTGAAATAACCTCTGAAAGAGGAACTTGGTTAGGTACCTTCTGAGGCGGAAAGAACCAGCTGTGGAATGTGTGTCAGTTAGGGTGTGGAAAGTCCCCAGGCTCCCCAGCAGGCAGAAGTATGCAAAGCATGCATCTCAATTAGTCAGCAACCAGGTGTGGAAAGCCCCAGGCTCCCCAGCAGGCAGAATATGCAAGCTGCTTTCATATTTAAGAAAACAAAAATCGCCGCCCCAAATCCGCGCATCCCCGCCAAA


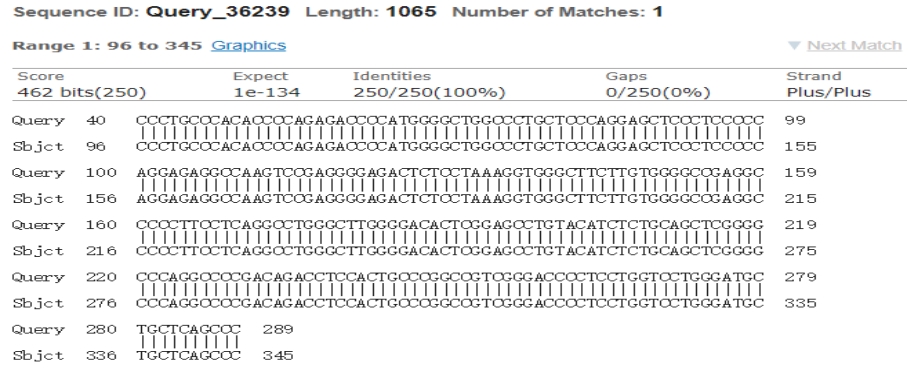


Correct

10.5.2 >Pmir-41WT-18-PmirGLO-F_H09

TCCGGATGGCGCCGCAAGATCCGCGAGATTCTCATTAAGGCCAAGAAGGGCGGCAAGATCGCCGTGTAATTCTAGTTGTTTAAACGAGCTCGCTAGCCCTGCCCACACCCCAGAGACCCCATGGGGCTGGCCCTGCTCCCAGGAGCTCCCTCCCCCAGGAGAGGCCAAGTCCGAGGGGAGACTCTCCTAAAGGTGGGCTTCTTGTGGGGCCGAGGCCCCCTTCCTCAGGCCTGGGCTTGGGGACACTCGGAGCCTGTACATCTCTGCAGCTCGGGGCCCAGGCCCCGACAGACCTCCACTGCCCGGCCGTCGGGACCCCTCCTGGTCCTGGGATGCTGCTCAGCCCTCGAGTCTAGAGTCGACCTGCAGGCATGCAAGCTGATCCGGCTGCTAACAAAGCCCGAAAGGAAGCTGAGTTGGCTGCTGCCACCGCTGAGCAATAACTAGCATAACCCCTTGGGGCGGCCGCTTCGAGCAGACATGATAAGATACATTGATGAGTTTGGACAAACCACAACTAGAATGCAGTGAAAAAAATGCTTTATTTGTGAAATTTGTGATGCTATTGCTTTATTTGTAACCATTATAAGCTGCAATAAACAAGTTAACAACAACAATTGCATTCATTTTATGTTTCAGGTTCAGGGGGAGATGTGGGAGGTTTTTTTAAGCAAGTAAAACCTCTACAAATGTGGTAAAATCGAATTTTAACAAAATATTAACGCTTACAATTTCCTGATGCGGTATTTTCTCCTTACGCATCTGTGCGGTATTTCACACCGCATACGCGGATCTGCGCAGCACCATGGCCTGAAATAACCTCTGAAAGAGGAACTTGGTTAGGTACCTTCTGAGGCGGAAAGAACCAGCTGTGGAATGTGTGTCAGTTAGGGTGTGGAAAGTCCCCAGGCTCCCCAGCAGGCAGAAGTATGCAAAGCATGCATCTCAATTAGTCAGCAACCAGGTGTGGAAAGTCCCAGGCTCCCCGCAGGCAGAATTTGCAAGCTGCTTCATATATAGAACAAACCAAGATCCCCGCCCTAAATCCGGCCATCCGGCCCAAAT


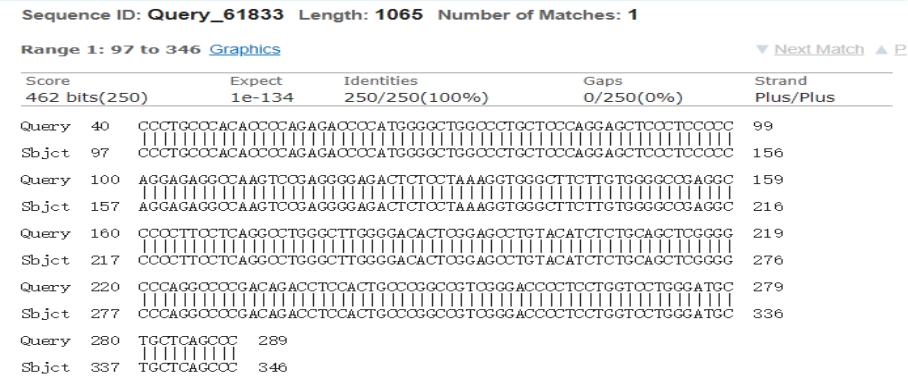


Correct

**10.6XM_005701596.3-WT**

GGGCGCAGACGGAGGCCGTCTGGGGTTCCTGGCTGTGCTGTGGGGGTGTGGAGGTGGTTTGTACCCGAGAACTCTAATAACACCAGCTGACACTTGTGTGATAA

10.6.1 >Pmir-96WT-10-PmirGLO-F_C10

CCCGAATGGAGCCGCAAGATCCGCGAGATTCTCATTAAGGCCAAGAAGGGCGGCAAGATCGCCGTGTAATTCTAGTTGTTTAAACGAGCTCGCTAGCGGGTTCCTGGCTGTGCTGTGGGGGTGTGGAGGTGGTTTGTACCCGAGAACTCTAATAACACCAGCTGACACTTGTGTGATAACTCGAGTCTAGAGTCGACCTGCAGGCATGCAAGCTGATCCGGCTGCTAACAAAGCCCGAAAGGAAGCTGAGTTGGCTGCTGCCACCGCTGAGCAATAACTAGCATAACCCCTTGGGGCGGCCGCTTCGAGCAGACATGATAAGATACATTGATGAGTTTGGACAAACCACAACTAGAATGCAGTGAAAAAAATGCTTTATTTGTGAAATTTGTGATGCTATTGCTTTATTTGTAACCA

TTATAAGCTGCAATAAACAAGTTAACAACAACAATTGCATTCATTTTATGTTTCAGGTTCAGGGGGAGATGTGGGAGGTTTTTTTAAGCAAGTAAAACCTCTACAAATGTGGTAAAATCGAATTTTAACAAAATATTAACGCTTACAATTTCCTGATGCGGTATTTTCTCCTTACGCATCTGTGCGGTATTTCACACCGCATACGCGGATCTGC

GCAGCACCATGGCCTGAAATAACCTCTGAAAGAGGAACTTGGTTAGGTACCTTCTGAGGCGGAAAGAACCAGCTGTGGAATGTGTGTCAGTTAGGGTGTGGAAAGTCCCCAGGCTCCCCAGCAGGCAGAAGTATGCAAAGCATGCATCTCAATTAGTCAGCAACCAGGTGTGGAAAGTCCCCAGGCTCCCCAGCAGGCAGAAGTAT

GCAAAGCATGCATCTCATTAGCAGGCAACCCATAGTCCCGCCCCTAACTCCGCCCATCCCGCCCCTAACTCCGCCCAGTTCCGCCCATTCTCCGCCCCATGGCTGACTAATTTTTTTATTTATGCAAAGGCCAAGGCCGCCTCGGCCTCTGAACTATTCCAGAATAGGAGGAGGTTTTTTGGAGGCCTAGGTTTTTGAAAAAGTTTGTTCTT


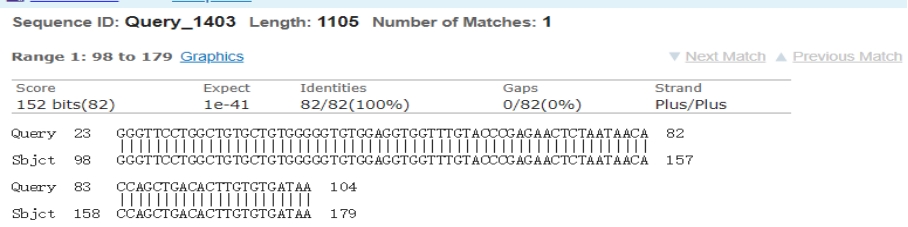
CTGAACAAAAGTTTCAAACAAAGGTTGAGCCCCCTGGGTTCCAGGGGGCAACCCGA

Correct

**11 XM_018063741.1-MU and XM_005701596.3-MU**

**11.1 Whole genome sequencing results**

11.1.1 XM_018063741.1-MU

GGGGACTGTGTCTGGACCGAGCTGTGCACTTGCACAGCACCCTGCCCACACCCCAGAGACCCCATGGGGCTGGCCCTGCTCCCAGGAGCTCCCTCCCCCAGGAGAGGCCAAGTCCGAGGGGAGACTCTCCTAAAGGTGGGCTTCTTGTGGGGCCGAGGCCCCCTTCCTCAGGCCTGGGCTTGGGGACACTCGGAGCCTGTACATCTCTGCAGCTCGGGGCCCAGGCCCCGACAGACCTCCACTGCCCGGCCGTCGGGACCCCTCCTGGTCCTGGTCGTAGTAGCAGCCCCTACCTCCGGC

11.1.1.1LA17943-2-XM_018063741.1-MU.seq

GGGGACTGTGTCTGGACCGAGCTGTGCACTTGCACAGCACCCTGCCCACACCCCAGAGACCCCATGGGGCTGGCCCTGCTCCCAGGAGCTCCCTCCCCCAGGAGAGGCCAAGTCCGAGGGGAGACTCTCCTAAAGGTGGGCTTCTTGTGGGGCCGAGGCCCCCTTCCTCAGGCCTGGGCTTGGGGACACTCGGAGCCTGTACATCTCTGCAGCTCGGGGCCCAGGCCCCGACAGACCTCCACTGCCCGGCCGTCGGGACCCCTCCTGGTCCTGGTCGTAGTAGCAGCCCCTACCTCCGGC


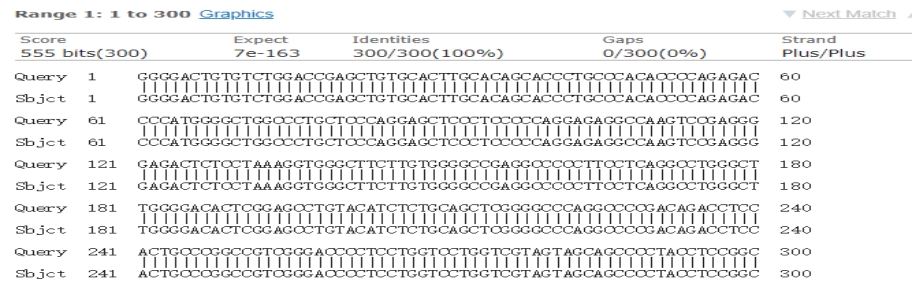


Correct

11.1.2 XM_005701596.3-MU

GGGCGCAGACGGAGGCCGTCTGGTTTGAAGTTATTGTAGTGGGGGGTGTGGAGGTGGTTTGTACCCGAGAACTCTAATAACACCAGCTGACACTTGTGTGATAA

11.1.2.1 LA17943-1-XM_005701596.3-MU.seq


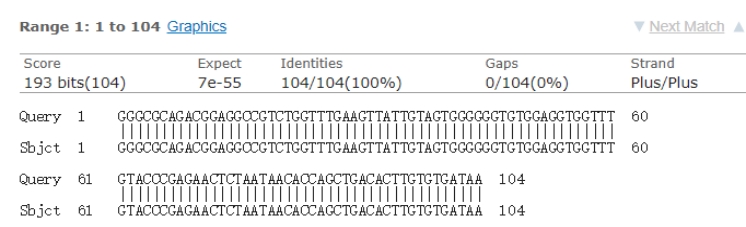
GGGCGCAGACGGAGGCCGTCTGGTTTGAAGTTATTGTAGTGGGGGGTGTGGAGGTGGTTTGTACCCGAGAACTCTAATAACACCAGCTGACACTTGTGTGATAA

Correct

Plasmid extraction is followed by the correct order.

**11.2 Plasmid extraction**

Use plasmid small extraction kit to extract plasmid, the operation steps refer to the instruction.

11.2.1 Plasmid purity and concentration detection

Take 2μL, use NanoDrop2000 to measure the purity and concentration of plasmid, the results are shown in the following table:

| **NO** | **泳道号** | **样本编号** | **样本类型** | **浓度（ng/μl）** | **OD260/280** |
| --- | --- | --- | --- | --- | --- |
| 1 | 1 | XM_018063741.1-MU | bacteria | 177 | 1.88 |
| 2 | 2 | XM_005701596.3-MU | bacteria | 214.5 | 1.85 |

11.2.2 Agarose gel electrophoresis diagram

1.5% agarose gel electrophoresis, 2μL was taken to identify the integrity of the plasmid. The results were shown as follows:


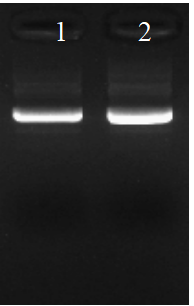


**11.3 PCR amplification**

PrimeSTAR^®^ HS DNA Polymerase is used for PCR reaction. The reaction system is as follows:

| Composition | Volume |
| --- | --- |
| Template（plasmid） | 200ng |
| Forward | 2.5 μL(10μM) |
| Reverse | 2.5 μL(10μM) |
| 5×PrimeSTAR Buffer (Mg^2+^ plus) | 20 μL |
| dNTP | 5 μL |
| PrimeSTAR HS DNA Polymerase (2.5 U/μl) | 0.5μL |
| H_2_O up to | 100 μL |

1. PCR reaction： 98℃ 10s, 60℃ 15s, 72℃ 40s, 40 cycle，72℃ 5min,4℃ ∞。
2. After PCR,1.5% Agarose was used to identify the size of the target fragment.

**11.4 PCR amplification**


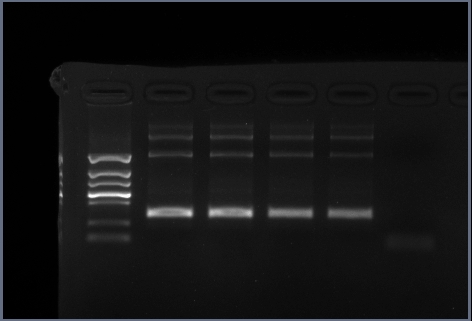


M 1 2 3 4 H_2_O

M:Marker;

1-4：41MU amplification

1500bp

500bp

200bp

100bp

400bp

700bp

900bp


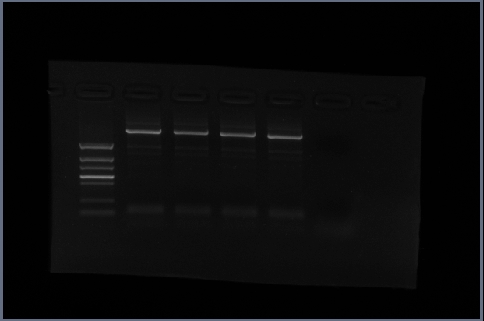


M 1 2 3 4 H_2_O

M:Marker;

1-4：96MU amplification

100bp

400bp

500bp

700bp

900bp

1500bp

200bp

The amplified bands were all in line with the target band size and were cut and recycled.


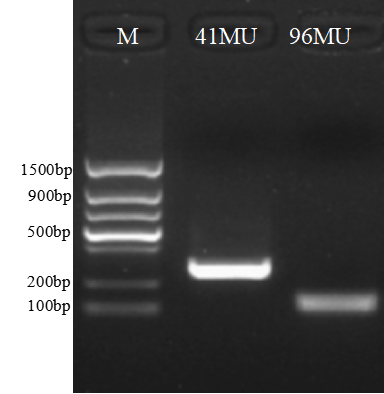


**11.5 Double enzyme digestion**

| Composition | Volume |
| --- | --- |
| Recycled product of PCR /pmirGLO | 6 μg |
| 10×rCutSmart Buffer | 10 μL |
| NheI-HF | 6 μL |
| XhoI | 6 μL |
| H_2_O up to | 100 μL |

Each were cut for two tubes, react overnight at 37℃, and cut glue for recycling.


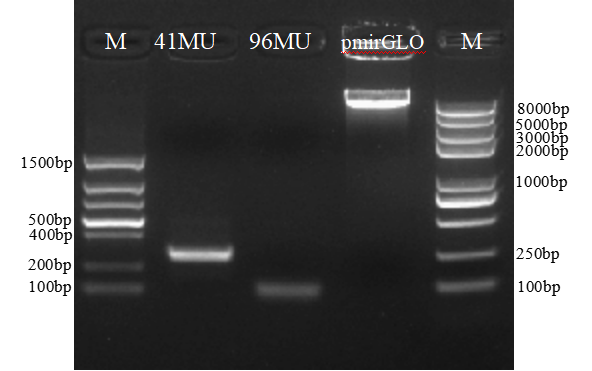


**11.6 ligase**

| Composition | Volume |
| --- | --- |
| Double enzyme digestion to recover the target fragment（<20ng/μL） | 7μL |
| Double enzyme digestion recovery pmirGLO（20ng/μL） | 1 μL |
| T4 DNA 连接酶 | 1 μL |
| 10×T4 Buffer | 1 μL |

16℃ connection reaction overnight, transformation, drawing board (refer to 8 for specific steps).

**11.7 Colony PCR validation and sequencing**

Single colony was selected for PCR amplification verification. PCR amplification procedure:98℃ 10s, 60℃ 15s, 72℃ 40s, 30 cycle，72℃ 5min,4℃ ∞. Correct clones were selected for bacterial liquid sequencing verification. The sequencing company was Genewiz, China, and the sequencing primer was pmirGLO-F（41MU：1/34；96MU：1/34）。


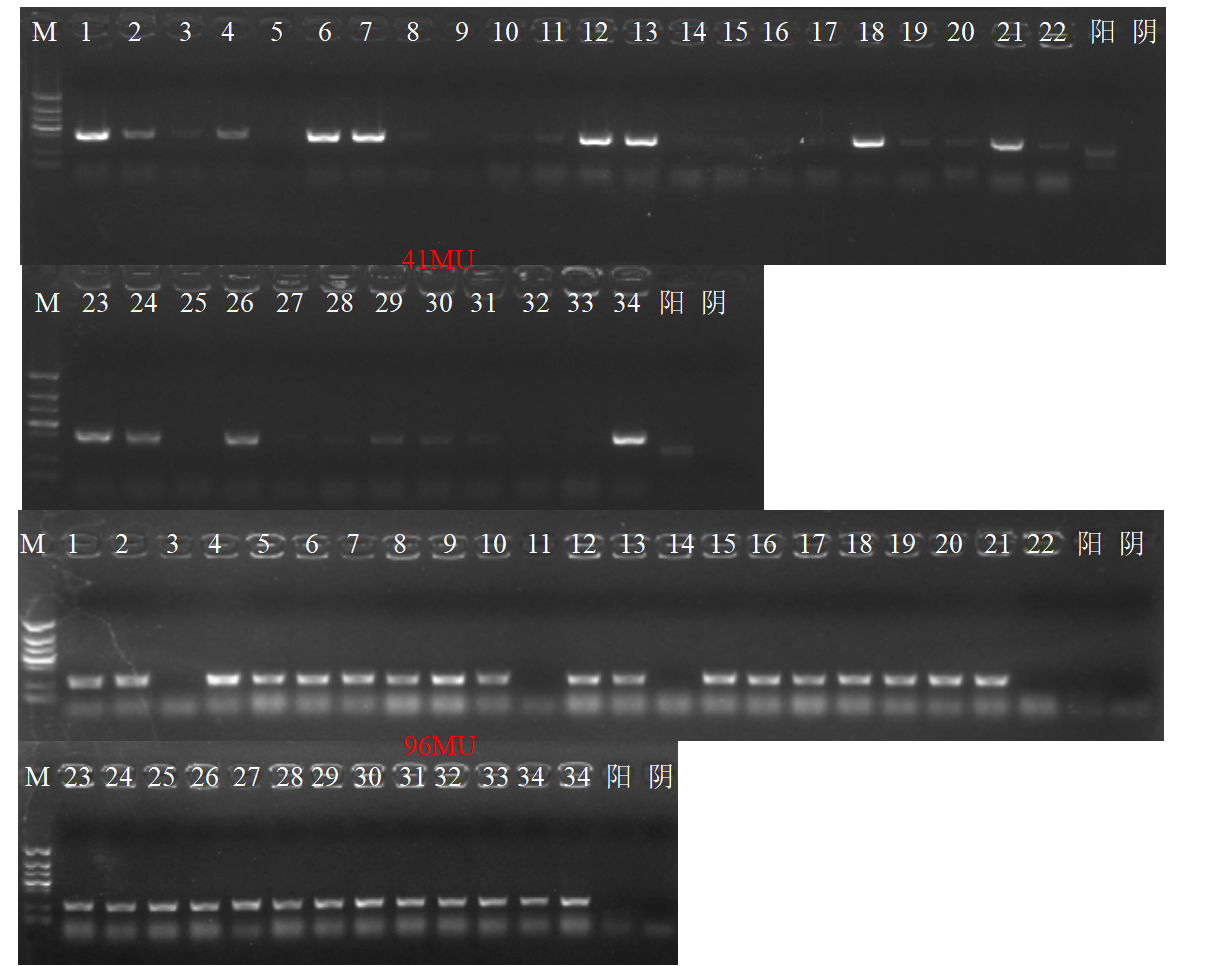


**11.8 Sequencing**

11.8.1 XM_005701596.3-MU

GGGCGCAGACGGAGGCCGTCTGGTTTGAAGTTATTGTAGTGGGGGGTGTGGAGGTGGTTTGTACCCGAGAACTCTAATAACACCAGCTGACACTTGTGTGATAA

11.8.2 >96MU-1-pmirGlo-F_C12

CCCCGGATGGAGCCGCAAGATCCGCGAGATTCTCATTAAGGCCAAGAAGGGCGGCAAGATCGCCGTGTAATTCTAGTTGTTTAAACGAGCTCGCTAGCGGTTTGAAGTTATTGTAGTGGGGGGTGTGGAGGTGGTTTGTACCCGAGAACTCTAATAACACCAGCTGACACTTGTGTGATAACTCGAGTCTAGAGTCGACCTGCAGGCATGCAAGCTGATCCGGCTGCTAACAAAGCCCGAAAGGAAGCTGAGTTGGCTGCTGCCACCGCTGAGCAATAACTAGCATAACCCCTTGGGGCGGCCGCTTCGAGCAGACATGATAAGATACATTGATGAGTTTGGACAAACCACAACTAGAATGCAGTGAAAAAAATGCTTTATTTGTGAAATTTGTGATGCTATTGCTTTATTTGTAACCATTATAAGCTGCAATAAACAAGTTAACAACAACAATTGCATTCATTTTATGTTTCAGGTTCAGGGGGAGATGTGGGAGGTTTTTTTAAGCAAGTAAAACCTCTACAAATGTGGTAAAATCGAATTTTAACAAAATATTAACGCTTACAATTTCCTGATGCGGTATTTTCTCCTTACGCATCTGTGCGGTATTTCACACCGCATACGCGGATCTGCGCAGCACCATGGCCTGAAATAACCTCTGAAAGAGGAACTTGGTTAGGTACCTTCTGAGGCGGAAAGAACCAGCTGTGGAATGTGTGTCAGTTAGGGTGTGGAAAGTCCCCAGGCTCCCCAGCAGGCAGAAGTATGCAAAGCATGCATCTCAATT
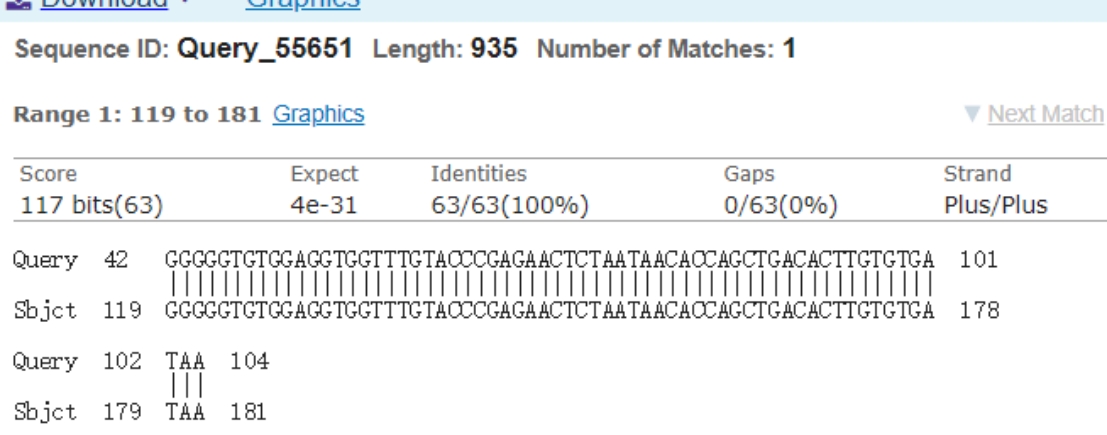
AGTCAGCAACCAGGTGTGGAAAGTCCCCAGGCTCCCCAGCAGGCAGAAGTATGCAAAGCATGCATCTCAATTAGTCAGCAACCATAGTCCCGCCCCTAACTCCGCCCATCCCGCCCCTAACTCCGCCCAGTTCCGCCCATTCTCCGCC

Correct

11.8.3 >96MU-34-pmirGlo-F_D12

CCCCGAATGGAGCCGCAAGATCCGCGAGATTCTCATTAAGGCCAAGAAGGGCGGCAAGATCGCCGTGTAATTCTAGTTGTTTAAACGAGCTCGCTAGCGGTTTGAAGTTATTGTAGTGGGGGGTGTGGAGGTGGTTTGTACCCGAGAACTCTAATAACACCAGCTGACACTTGTGTGATAACTCGAGTCTAGAGTCGACCTGCAGGCATGCAAGCTGATCCGGCTGCTAACAAAGCCCGAAAGGAAGCTGAGTTGGCTGCTGCCACCGCTGAGCAATAACTAGCATAACCCCTTGGGGCGGCCGCTTCGAGCAGACATGATAAGATACATTGATGAGTTTGGACAAACCACAACTAGAATGCAGTGAAAAAAATGCTTTATTTGTGAAATTTGTGATGCTATTGCTTTATTTGTAACCATTATAAGCTGCAATAAACAAGTTAACAACAACAATTGCATTCATTTTATGTTTCAGGTTCAGGGGGAGATGTGGGAGGTTTTTTTAAGCAAGTAAAACCTCTACAAATGTGGTAAAATCGAATTTTAACAAAATATTAACGCTTACAATTTCCTGATGCGGTATTTTCTCCTTACGCATCTGTGCGGTATTTCACACCGCATACGCGGATCTGCGCAGCACCATGGCCTGAAATAACCTCTGAAAGAGGAACTTGGTTAGGTACCTTCTGAGGCGGAAAGAACCAGCTGTGGAATGTGTGTCAGTTAGGGTGTGGAAAGTCCCCAGGCTCCCCAGCAGGCAGAAGTATGCAAAGCATGCATCTCAATTAGTCAGCAACCAGGTGTGGAAAGTCCCCAGGCTCCCCAGCAGGCAGAAGTATGCAAAGCATGCATCTCAATTAGTCAGCAACCATAGTCCCGCCCCTAACTCCGCCCATCCCGCCCCTAACTCCGCCCAGTTCCGCCCATTCTCCGCCCCATGG


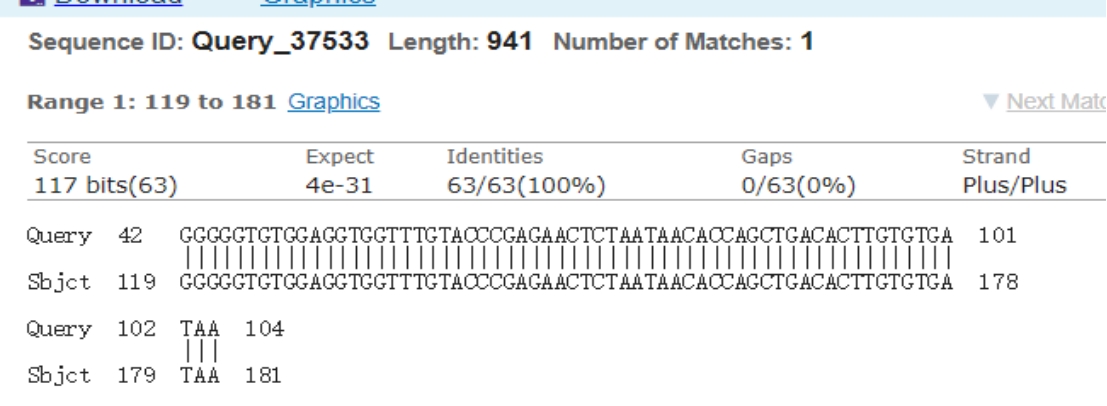


Correct

11.8.4 XM_018063741.1-MU

GGGGACTGTGTCTGGACCGAGCTGTGCACTTGCACAGCACCCTGCCCACACCCCAGAGACCCCATGGGGCTGGCCCTGCTCCCAGGAGCTCCCTCCCCCAGGAGAGGCCAAGTCCGAGGGGAGACTCTCCTAAAGGTGGGCTTCTTGTGGGGCCGAGGCCCCCTTCCTCAGGCCTGGGCTTGGGGACACTCGGAGCCTGTACATCTCTGCAGCTCGGGGCCCAGGCCCCGACAGACCTCCACTGCCCGGCCGTCGGGACCCCTCCTGGTCCTGGTCGTAGTAGCAGCCCCTACCTCCGGC

11.8.5 >41MU-1-pmirGlo-F_E12

CCCCGAATGGAGCCGCAAGATCCGCGAGATTCTCATTAAGGCCAAGAAGGGCGGCAAGATCGCCGTGTAATTCTAGTTGTTTAAACGAGCTCGCTAGCCCTGCCCACACCCCAGAGACCCCATGGGGCTGGCCCTGCTCCCAGGAGCTCCCTCCCCCAGGAGAGGCCAAGTCCGAGGGGAGACTCTCCTAAAGGTGGGCTTCTTGTGGGGCCGAGGCCCCCTTCCTCAGGCCTGGGCTTGGGGACACTCGGAGCCTGTACATCTCTGCAGCTCGGGGCCCAGGCCCCGACAGACCTCCACTGCCCGGCCGTCGGGACCCCTCCTGGTCCTGGTCGTAGTAGCTCGAGTCTAGAGTCGACCTGCAGGCATGCAAGCTGATCCGGCTGCTAACAAAGCCCGAAAGGAAGCTGAGTTGGCTGCTGCCACCGCTGAGCAATAACTAGCATAACCCCTTGGGGCGGCCGCTTCGAGCAGACATGATAAGATACATTGATGAGTTTGGACAAACCACAACTAGAATGCAGTGAAAAAAATGCTTTATTTGTGAAATTTGTGATGCTATTGCTTTATTTGTAACCATTATAAGCTGCAATAAACAAGTTAACAACAACAATTGCATTCATTTTATGTTTCAGGTTCAGGGGGAGATGTGGGAGGTTTTTTTAAGCAAGTAAAACCTCTACAAATGTGGTAAAATCGAATTTTAACAAAATATTAACGCTTACAATTTCCTGATGCGGTATTTTCTCCTTACGCATCTGTGCGGTATTTCACACCGCATACGCGGATCTGCGCAGCACCATGGCCTGAAATAACCTCTGAAAGAGGAACTTGGTTAGGTACCTTCTGAGGCGGAAAGAACCAGCTGTGGAATGTGTGTCAGTTAGGGTGTGGAAAGTCCCCAGGCTCCCCAGCAGGCAGAAGTATGCAAAGCA


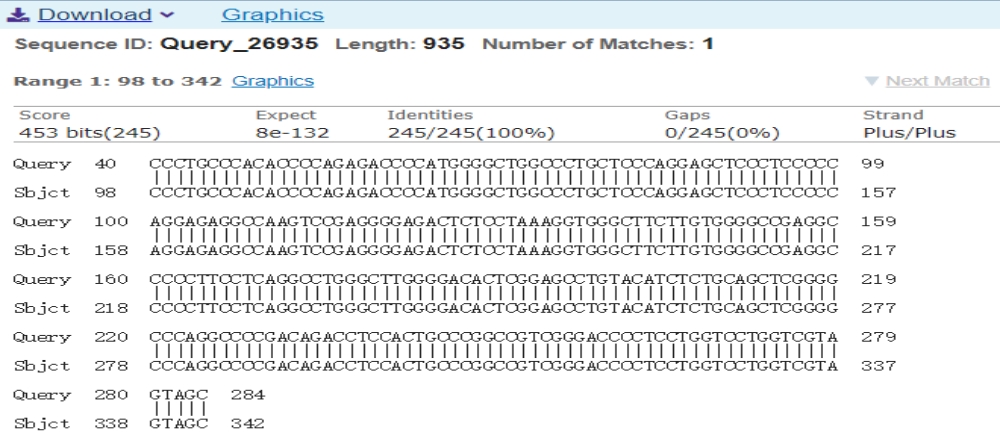


Correct

11.8.6 >41-MU-34-pmirGlo-F_H03

TCCGGGATGGCCCCGCAAGAACCGCGAGAATTCTCATTAAGGCCAAGAAGGGCGGCAAGATCGCCGTGTAATTCTAGTTGTTTAAACGAGCTCGCTAGCCCTGCCCACACCCCAGAGACCCCATGGGGCTGGCCCTGCTCCCAGGAGCTCCCTCCCCCAGGAGAGGCCAAGTCCGAGGGGAGACTCTCCTAAAGGTGGGCTTCTTGTGGGGCCGAGGCCCCCTTCCTCAGGCCTGGGCTTGGGGACACTCGGAGCCTGTACATCTCTGCAGCTCGGGGCCCAGGCCCCGACAGACCTCCACTGCCCGGCCGTCGGGACCCCTCCTGGTCCTGGTCGTAGTAGCTCGAGTCTAGAGTCGACCTGCAGGCATGCAAGCTGATCCGGCTGCTAACAAAGCCCGAAAGGAAGCTGAG

TTGGCTGCTGCCACCGCTGAGCAATAACTAGCATAACCCCTTGGGGCGGCCGCTTCGAGCAGACATGATAAGATACATTGATGAGTTTGGACAAACCACAACTAGAATGCAGTGAAAAAAATGCTTTATTTGTGAAATTTGTGATGCTATTGCTTTATTTGTAACCATTATAAGCTGCAATAAACAAGTTAACAACAACAATTGCATTCATTTTATGTTTCAGGTTCAGGGGGAGATGTGGGAGGTTTTTTTAAGCAAGTAAAACCTCTACAAATGTGGTAAAATCGAATTTTAACAAAATATTAACGCTTACAATTTCCTGATGCGGTATTTTCTCCTTACGCATCTGTGCGGTATTTCACACCGCATACGCGGATCTGCGCAGCACCATGGCCTGAAATAACCTCTGAAAGAGGAACTTG

GTTAGGTACCTTCTGAGGCGGAAAGAACCAGCTGTGGAATGTGTGTCAGTTAGGGTGTGGAAAGTCCCCAGGCTCCCCAGCAGGCAGAAGTATGCAAAGCATGCATCTCAATTAGTCAGCAACCAGGTGTGGAAAGTCCCCAGGCTCCCCAGCAGGCAGAAGTATGCAAAGCATGCATCTCAATTAGTCAGCAACCATAGTCCCGGC
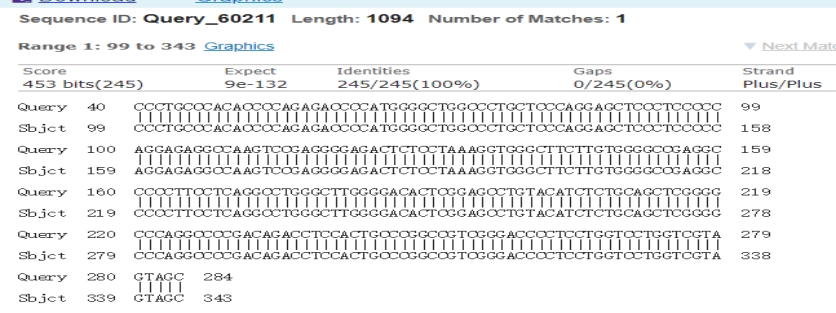
CCTAACTCCGCCCATCCCGCCCTAACTCCGCCAAGTTCCGCCATTTTCCGCC

Correct

**12 Plasmid extraction**

Use endotoxin-free kit to extract plasmid, refer to the instructions.

**12.1 Plasmid purity and concentration detection**

Take 2μL, use NanoDrop2000 to measure the purity and concentration of plasmid, the results are shown in the following table:

| **NO** | **Electrophoresis No.** | **Sample No.** | **Sample type** | **Consentration（ng/μl）** | **OD260/280** |
| --- | --- | --- | --- | --- | --- |
| 1 | 1 | pmirGLO-XM_005676701.3-WT-14 | bacteria | 573 | 1.88 |
| 2 | 2 | pmirGLO-XM_005676701.3-MU-10 | bacteria | 686.3 | 1.85 |
| 3 | 3 | pmirGLO-XM_018064739.1-WT-5 | bacteria | 529.1 | 1.87 |
| 4 | 4 | pmirGLO-XM_018064739.1-MU-24 | bacteria | 648.2 | 2.01 |
| 5 | 5 | pmirGLO-XM_018063741.1-WT-18 | bacteria | 511.6 | 1.81 |
| 6 | 6 | pmirGLO-XM_018063741.1-MU-1 | bacteria | 816.2 | 1.84 |
| 7 | 7 | pmirGLO-XM_005701596.3-WT-10 | bacteria | 800.8 | 1.86 |
| 8 | 8 | pmirGLO-XM_005701596.3-MU-34 | bacteria | 477 | 1.85 |

**12.2 Agarose gel electrophoresis diagram**

1.5% agarose gel electrophoresis, 2μL was taken to identify the integrity of the plasmid. The results were shown as follows:


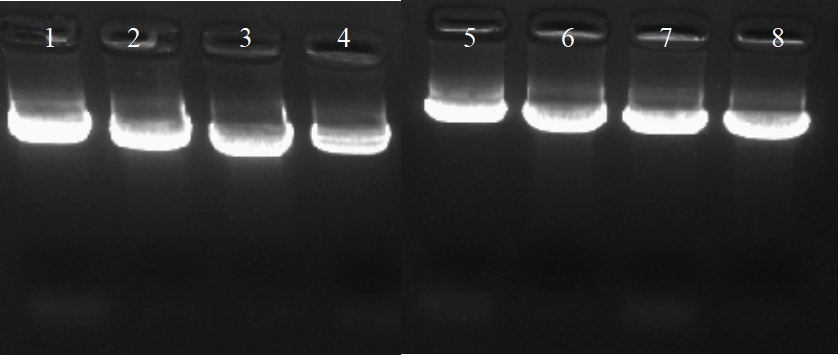

Supplement: S4 File — (DOCX) [file pone.0282772.s004.docx]
