# Supplementary material for: Transcriptomes reveal microRNAs and mRNAs in different photoperiods influencing cashmere growth in goat
Source: PLoS One. 2023 Mar 17;18(3):e0282772. doi: 10.1371/journal.pone.0282772 (PMC10022811; doi:10.1371/journal.pone.0282772)
Supplement: Attachment — Submitted filename: PONE-D-22-12093.docx [file pone.0282772.s006.docx]

In the manuscript “Transcriptomes reveal microRNAs and mRNAs in different photoperiods influencing cashmere growth in goat” the author wanted to show the mechanism behind the effect of photoperiod on cashmere growth in the inner Mongolian Arbas goat. Although the overall study design and results are satisfactory, but I have couple of concern about the manuscript.

1. Since the photoperiod shortening co-occur with winter or low temperature, how do author nullify the effect of temperature as they used the same temperature of outside in the goat shed? It could be the effect is due to low temperature and not photoperiod.
2. There is no statistical test done for Fig 4? Also, author didn’t wanted to explain the results of that experiment. How much relevant is using 293T cells for assessing gene expression of skin epithelial cells.
